# Inhibitors of phosphoinositide 3-kinase (PI3K) and phosphoinositide 3-kinase-related protein kinase family (PIKK)

**DOI:** 10.1080/14756366.2023.2237209

**Published:** 2023-07-24

**Authors:** Xueqin Huang, Li You, Eugenie Nepovimova, Miroslav Psotka, David Malinak, Marian Valko, Ladislav Sivak, Jan Korabecny, Zbynek Heger, Vojtech Adam, Qinghua Wu, Kamil Kuca

**Affiliations:** a College of Life Science, Yangtze University, Jingzhou, China; b College of Physical Education and Health, Chongqing College of International Business and Economics, Chongqing, China; c Department of Chemistry, Faculty of Science, University of Hradec Kralove, Czech Republic; d Biomedical Research Center, University Hospital Hradec Kralove, Hradec Kralove, Czech Republic; e Faculty of Chemical and Food Technology, Slovak University of Technology in Bratislava, Bratislava, Slovakia; f Department of Chemistry and Biochemistry, Mendel University in Brno, Brno, Czech Republic

**Keywords:** PI3K, PIKK, AKT, inhibitors, anticancer therapy

## Abstract

Phosphoinositide 3-kinases (PI3K) and phosphoinositide 3-kinase-related protein kinases (PIKK) are two structurally related families of kinases that play vital roles in cell growth and DNA damage repair. Dysfunction of PIKK members and aberrant stimulation of the PI3K/AKT/mTOR signalling pathway are linked to a plethora of diseases including cancer. In recent decades, numerous inhibitors related to the PI3K/AKT/mTOR signalling have made great strides in cancer treatment, like copanlisib and sirolimus. Notably, most of the PIKK inhibitors (such as VX-970 and M3814) related to DNA damage response have also shown good efficacy in clinical trials. However, these drugs still require a suitable combination therapy to overcome drug resistance or improve antitumor activity. Based on the aforementioned facts, we summarised the efficacy of PIKK, PI3K, and AKT inhibitors in the therapy of human malignancies and the resistance mechanisms of targeted therapy, in order to provide deeper insights into cancer treatment.

## Introduction

Phosphoinositide 3-kinase (PI3K) is a family of protein and lipid kinases, which phosphorylates the 3′-OH position on the inositol ring of phosphatidylinositol (4,5)-bisphosphate (PtdIns(4,5)P2) to generate PtdIns(3,4,5)P3^1^. PtdIns(3,4,5)P3 (PIP3) serves as a docking point to recruit proteins to the vicinity of the plasma membrane by connecting to the protein’s pleckstrin homologous (PH) domain, with the most important protein being the AKT family of serine threonine kinases^2^. AKT, also referred to as protein kinase B (PKB), is a key signalling hub in the PI3K pathway. Its activation and overexpression can further regulate multiple downstream signalling targets and are closely correlated with resistance to cancer radiotherapy or chemotherapy treatments^3^. PIKK and PI3K are two closely related families with similar kinase domains. PIKK is composed of six atypical serine/threonine protein kinases, including ataxia telangiectasia mutated (ATM), ataxia telangiectasia-and-rad3-related (ATR), DNA-dependent protein kinase (DNA-PK), mammalian target of rapamycin (mTOR), suppressor of morphogenesis in genitalia (SMG-1), and transformation/transcription associated protein (TRAAP)^4^. PIKK members ATM, ATR, and DNA-PK associated with DNA damage response (DDR) are closely related to maintaining genomic stability^5^. mTOR is the main mediator of PI3K/AKT signalling, which can regulate cell growth, survival, and metabolism. Therefore, its activation can promote tumour growth and metastasis^6^. SMG-1 was first identified for RNA monitoring through nonsense-mediated mRNA decay (NMD), but it was later discovered that SMG-1 is independent of NMD itself and inhibits DNA repair through non-homologous end connections^7^^,^^8^. There is limited research on TRAAP, which is the only member of the PIKK kinase family that lacks all catalytic residues and can activate gene expression in important processes^9^^,^^10^. In summary, PI3K and PIKK family kinases play crucial roles in regulating various responses, including cell proliferation, metabolism, survival, and gene stability, making them important targets for research.

The PI3K/AKT/mTOR signalling pathway is one of the key intracellular signalling pathways that can undergo abnormal activation in most cancers^11–13^. Mutations in genes such as *PIK3CA*, phosphatase and tension homologues (*PTEN*), *AKT1*, and tuberous sclerosis 1/2 (*TSC1/2*) can all lead to dysregulation of signalling pathways, which can drive important physiological processes such as tumour metabolism, proliferation, angiogenesis, and metastasis^14–17^. Therefore, targeting key molecules in the PI3K/AKT/mTOR pathway is a promising cancer treatment strategy. PIKK members ATM, ATR, and DNA-PK are closely related to DNA damage repair and have complex effects on cancer progression and treatment. On the one hand, genomic instability is a basic characteristic of cancer, and defects in the DNA repair pathway can lead to the formation and development of cancer cells^18^. On the other hand, this also exposes the fragility of these tumour cells, making them more susceptible to DNA damage agents^19^. A method called "synthetic lethal" can selectively kill tumour cells by using drugs to inhibit the remaining DNA repair pathways^20^. For example, in ATM-deficient xenograft models, ATR inhibition can induce synthesis lethality and exert chemical sensitisation effects^21^. It is worth noting that inhibiting DNA repair can lead to the accumulation of cytoplasmic DNA and induce type I interferon (IFN) in a STING (stimulator of interferon genes) -dependent manner, which is beneficial for activating the innate immune response and promoting tumour eradication^22^. Given the limited research of other members of PIKK and the prominent role of ATM, ATR, and DNA-PK in DNA damage reactions, this article mainly introduces the mechanisms of action of ATM, ATR, and DNA-PK. In summary, the abnormal activation of the PI3K/AKT/mTOR signalling pathway and the dysfunction of PIKK members in DNA damage repair are closely related to the occurrence of cancer and drug resistance during treatment. Therefore, the development of targeted drugs has become an effective approach for anti-tumour therapy.

In this review, we introduce the activation of the PI3K/AKT/mTOR signalling pathway in cancer and the mechanisms by which PIKK members ATM, ATR, and DNA-PK repair DNA damage. In addition, we summarise the efficacy and achievements of PIKK, PI3K, and AKT inhibitors in various human malignancies. We focused particularly on the drugs that entered clinical trials and were already approved for marketing, as well as the efficacy of these drugs in combination with other anticancer drugs applied in therapy of different types of cancers. We also discuss tumour resistance to the inhibitors and propose new approaches to improve antitumor activity by developing multi-targeted drugs, utilising drug delivery systems, and optimising combination regimens. Taken together, this article reviews the clinical research stages and efficacy of PIKK, PI3K, and AKT inhibitors as antitumor drugs in order to provide new perspectives and prospects for cancer treatment.

## Phosphoinositide 3-kinase (PI3K)

PI3Ks are an evolutionarily conserved family of lipid kinases classified into three types according to their structure and function^23^. Class, I PI3Ks consist of a heterodimer of a regulatory and a catalytic subunit (p110α, p110β, p110δ, and p110γ), which is most associated with cancer cell growth^24^^,^^25^. Class I PI3Ks contain two categories, namely IA and IB. The catalysed subunits of category IA, p110α, p110β, and p110δ, are encoded by *PIK3CA*, *PIK3CB*, and *PIK3CD* genes, respectively, and the regulatory subunit is usually p85 type, including p85α (and its splicing variants p55α and p50α), p85β, p55γ^26^. The catalytic subunit p110γ of class IB is encoded by *PIK3CG* and its regulatory subunits are p101, p84 (p84 or p87)^26^. However, the p110α subunit mutation in *PIK3CA* is considered one of the most prevalent mutations involved in cancer development^27^. The most important mutation sites of p110α are the helical domain E542K and E545K sites, as well as the kinase domain H1047R, all of which have a synergistic effect on cellular transformation^28^. PI3Kα and β isoforms can be found in many different tissues, while δ and γ are mainly present in the immune system^29^^,^^30^. The other two PI3K classes have received less attention in terms of cancer research. Class II PI3K has three isomers, namely *PI3KC2α*, *PI3KC2β* and *PI3KC2γ*^31^. At present, the physiological effects of class II PI3K are not fully understood, but they are known to affect cellular activities such as glucose transport, endocytosis, cell migration, and survival^32^^,^^33^. The member of Class III PI3K is vacuolar protein sorting 34 (VPS34), which plays a key role in regulating intracellular transport, autophagy, and heart and liver function^34^.

**Figure 1.**
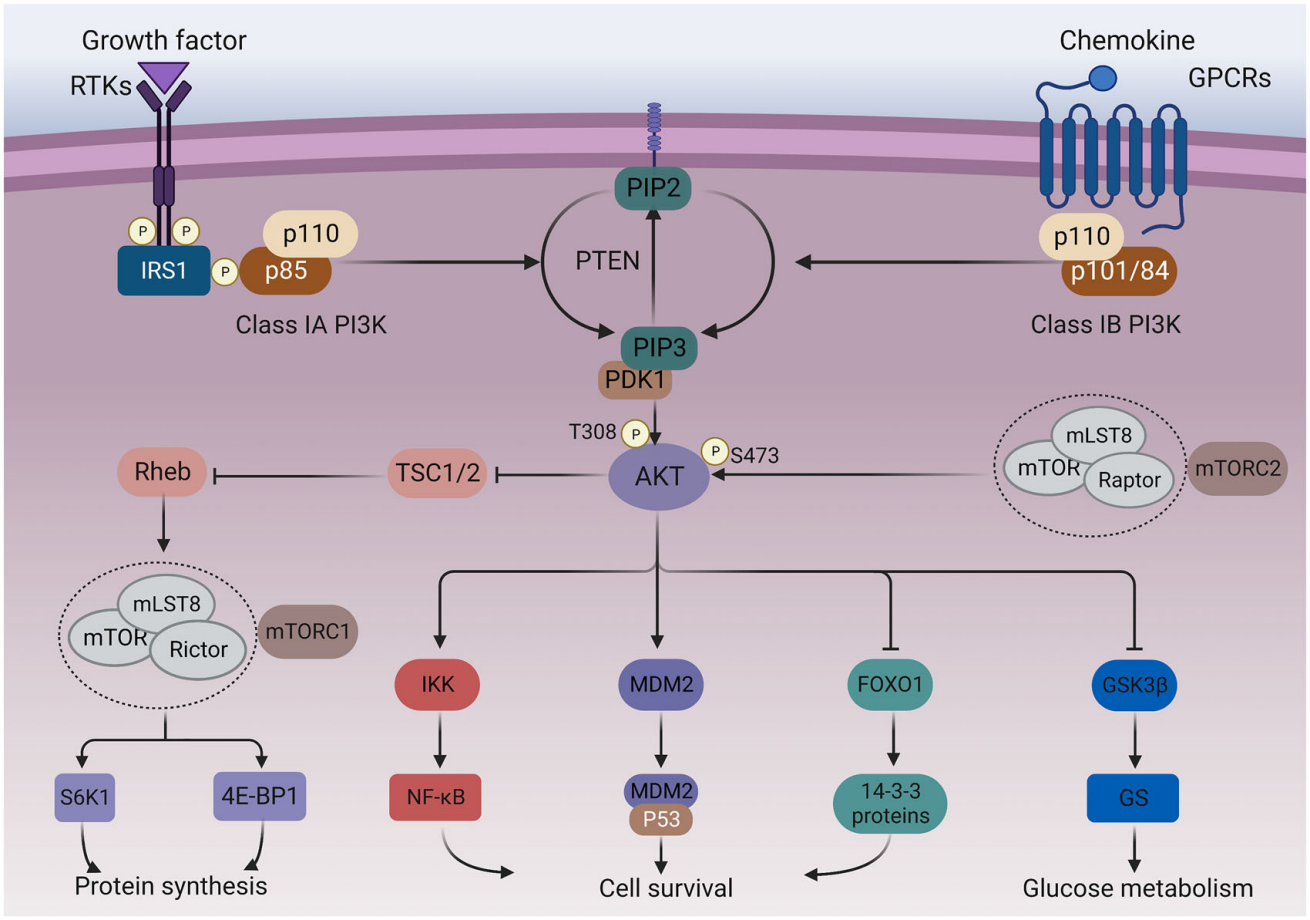
PI3K/AKT/mTOR signalling pathway. Growth factors bind to tyrosine kinase receptors (RTK) or G protein-coupled receptors (GPCR), activating PI3K heterodimers of the IA and IB families, respectively. Activated PI3K phosphorylates PIP2 into PIP3, while PTEN dephosphorylates PIP3. The activation of PIP3 attracts PDK1 and AKT to the plasma membrane. AKT can be phosphorylated by PDK1 at the T308 site, while mTORC2 phosphorylates the S473 site. Afterwards, the activated AKT can activate multiple downstream targets. The phosphorylation of tuberous sclerosis 2 (TSC2) by AKT can inhibit the TSC1/TSC2 complex, leading to indirect activation of mTORC1 by blocking the negative regulation of the Ras homolog (Rheb) by TSC1/2. S6 kinase 1 protein (S6K1) and eucaryotic initiation factor 4E-binding protein (4E-BP1) are downstream targets of mTORC1, which can control protein synthesis. In addition, AKT can also phosphorylate IKK, MDM2, FoxO1 to regulate cell survival, and GSK3β to regulate glucose metabolism.

PI3K is a meaningful transduction target in signalling pathways. It is worth noting that class IA can be triggered by various receptor tyrosine kinases or the binding of Ras proteins to the p110 subunit, whereas Class IB can be activated by G protein-coupled receptors^35^^,^^36^. After activation, the active PI3K phosphorylates phosphatidylinositol-4,5-bisphosphate to PIP3^37^. However, PTEN in turn can dephosphorylate PIP3^38^. After binding to the PH domain of the AKT, PIP3 translocates the protein to the vicinity of the plasma membrane^39^. Subsequently, the recruited AKT needs to be activated by the phosphorylation of two residues, T308 and S473, through phosphoinositol-dependent protein kinase 1 (PDK1) and mTOR complex 2 (mTORC2), respectively^39^^,^^40^. Activated AKT can activate and modulate a series of downstream targets, thereby significantly regulating tumour cell growth and proliferation, metastasis and invasion, and chemotherapy resistance^41^. The PI3K/AKT/mTOR signalling pathway is schematised in Figure 1.

## PI3K inhibitors

In the early stages of research and development, PI3K-targeted drugs were mainly composed of a series of pan-PI3K (pan-PI3K) inhibitors, with Wortmannin and LY294002 being prototypes of PI3K inhibitors^42^. Non-selective pan-PI3K inhibitors target all PI3K subtypes and may produce strong side effects, making subtype-specific PI3K inhibitors one of the hotspots in drug development^43^. In addition, dose limitations may also be a problem in the development process of PI3K inhibitors. Due to the fact that the PI3K signalling pathway is also indispensable in normal cells (with only low expression), high-dose medication is bound to affect the normal functions of other cells, leading to serious side effects that can be unbearable for patients and interrupting treatment^44^. At present, researchers are committed to developing a series of novel inhibitors with significant efficacy, low toxicity, and a few side effects. PI3K inhibitors can be subdivided into pan-PI3K inhibitors, isoform-specific inhibitors, and dual PI3K/mTOR inhibitors (Table 1).

**Table 1.** A summary of major PI3K inhibitors.

Inhibitor	Compound	Inventor	IC_50_ (nmol/L)	Phase	Clinical
Pan-PI3K inhibitors	Buparlisib(BKM120)	Novartis	PI3Kα/β/δ/γ: 52/166/116/262	III	NCT01610284
	Pictilisib(GDC-0941)	Genentech	PI3Kα/β/δ/γ: 3/33/3/75	II	NCT01740336
	Sonolisib(PX-866)	Oncothyreon	PI3Kα/δ/γ: 0.1/2.9/1	II	NCT01252628
	Pilaralisib(XL147)	Exelixis and Sanof	PI3Kα/β/δ/γ: 39/383/36/23	II	NCT01013324
Isoform-specific inhibitors	Alpelisib(BYL719)	Novartis	PI3Kα: 5	Approved	
	Inavolisib(GDC-0077)	Genentech	PI3Kα: 0.038	III-Ongoing	NCT04191499
	GSK2636771	GlaxoSmithKline	PI3Kβ: 5.2	II	NCT03131908
	Idelalisib	Gilead	PI3Kδ: 2.5	Approved	
	Umbralisib	TG Therapeutics	PI3Kδ: 22.2	Approved	
Dual PI3K / mTOR or dual PI3K inhibitors	Dactolisib(BEZ-235)	Novartis	PI3Kα/β/δ/γ: 4/75/7/5 mTOR: 20.7	Terminated	
	Omipalisib(GSK2126458)	GlaxoSmithKline	PI3Kα/β/δ/γ:0.019/0.13/0.06/0.024 mTORC1/2: 0.18/0.3	I	NCT01248858
	Gedatolisib((PKI-587)	Pfizer	PI3Kα/γ: 0.4/5.4 mTOR: 1.6	II	NCT01420081
	Apitolisib(GDC-0980)	Genentech	PI3Kα/β/δ/γ: 5/27/7/14 mTOR: 17 (Ki)	II	NCT01442090
	Paxalisib(GDC-0084)	Kazia	PI3Kα/β/δ/γ: 2/46/3/10 (Kiapp) mTOR: 70 (Kiapp)	II-Ongoing	NCT03765983
	Voxtalisib(XL765)	Sanof	PI3Kα/β/δ/γ: 39/113/9/43 DNA-PK/mTOR: 150/157	II	NCT01403636
	Copanlisib	Bayer	PI3Kα/δ: 0.5/0.7	Approved	
	Duvelisib(IPI-145)	Infinity	PI3Kδ/γ: 2.5/27.4	Approved	
	Tenalisib(RP6530)	Rhizen Pharmaceuticals SA	PI3Kδ/γ: 25/33	II	NCT05021900

### Pan-PI3K inhibitors

**Figure 2.**
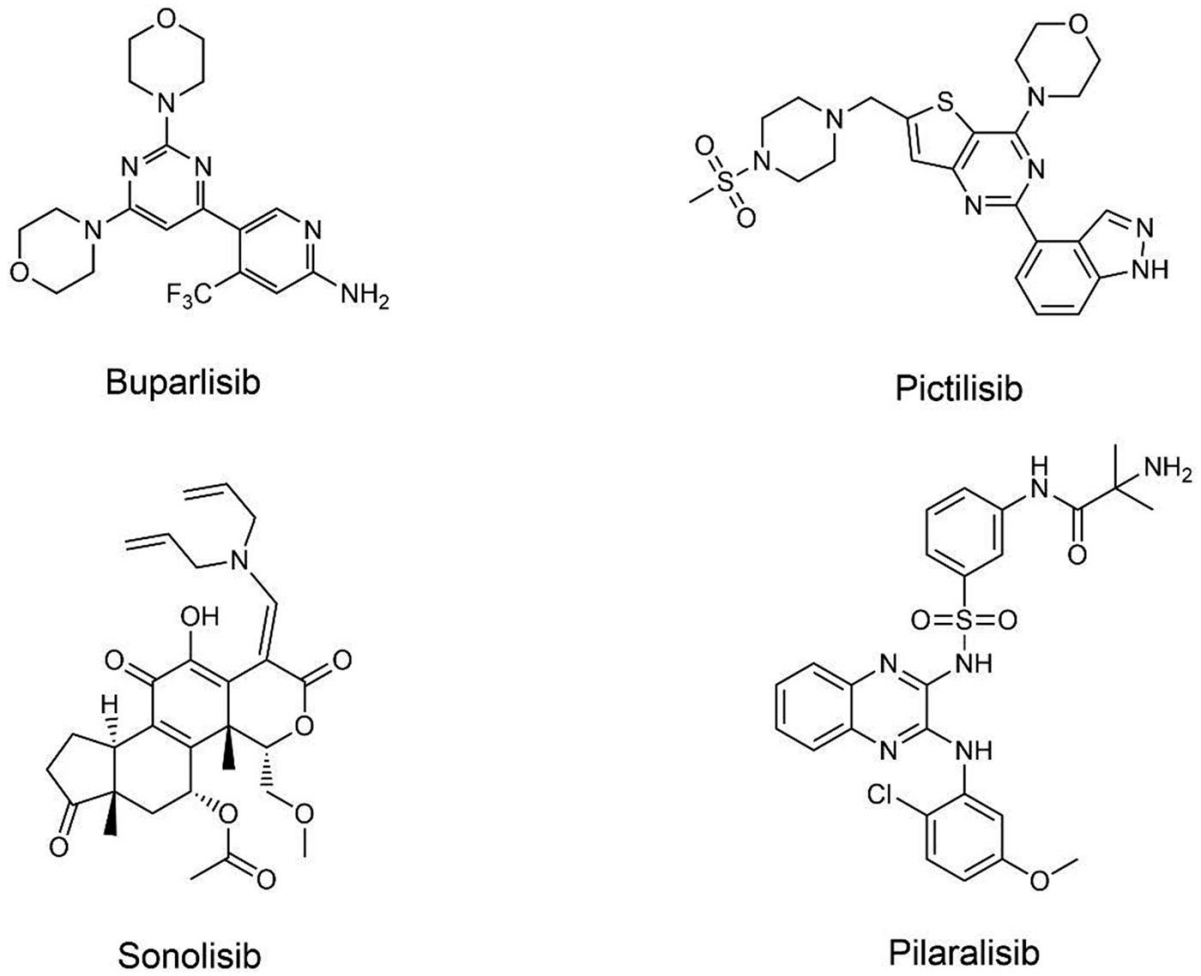
Structures of pan-PI3K inhibitors.

Pan-PI3K inhibitors are effective against all four isomers of Class I PI3K, leading to severe toxic side effects (Figure 2). Therefore, the development of a large number of related inhibitors has been discontinued. The current research on Pan-PI3K inhibitors entering clinical practice is as follows.

#### Buparlisib/NVP-BKM120/BKM120

Buparlisib is an oral pan-PI3K inhibitor belonging to a family of 2, 6-dimorpholinopyrimidine derivatives that inhibits 4 isoforms of PI3K *via* ATP competition^45^. Buparisib exhibits significant brain penetration in both *in vitro* and *in vivo* mouse models, indicating that it is a highly promising treatment option for intracranial tumors^46^. However, buparlisib showed low monotherapy efficacy in a phase II trial in patients with PI3K-activated glioblastoma (GBM), possibly due to insufficient inhibition of the PI3K pathway in the tumor^47^. As a result, later combined treatment trials were conducted. Unfortunately, buparlisib combined with radiotherapy and temozolomide is not safe. Therefore, Novartis has decided not to develop buparlisib in newly diagnosed glioblastoma^48^. At present, buparlisib appears to be a promising treatment for breast and head and HNSCC. In the phase III trial of buparlisib plus fulvestrant in hormone receptor-positive/epidermal growth factor receptor-2 negative (HR^+^/HER2^-^) advanced breast cancer, the combination of fulvestrant and buparlisib significantly prolonged progression-free survival, but the occurrence of severe side effects was higher than that of the placebo group, at 23% and 16%, respectively^49^. Buparlisib monotherapy was found to be ineffective in a phase II trial in HNSCC, but combination therapy had an unexpected synergistic anti-tumour effect without significantly increasing treatment toxicity^50^. At present, buparlisib has obtained the fast track approval granted by the FDA, and has been approved for a global multi-center phase III study (NCT04338399) of buparlisib plus paclitaxel for the treatment of HNSCC.

#### Pictilisib/GDC-0941

Pictilisib is an oral and potent PI3K inhibitor with significant activity against PI3K α/δ isoforms and moderate selectivity for PI3K β/γ, showing promising antitumor activity in preclinical xenograft models^51^. When pictilisib is coupled with anastrozole for treating patients with HR^+^/HER2^-^ early breast cancer, it can significantly inhibit tumour cell proliferation^52^, but there is no superior benefit when pictilisib is combined with paclitaxel or fulvestrant^53^^,^^54^. Additionally, the combination of pictilisib with erlotinib or cobimetinib demonstrated only minimal anticancer effects and tolerability in patients with solid tumors^55^^,^^56^. A study on pictilisib was conducted in Japan^57^. Four of twelve patients achieved stable disease in the first stage of monotherapy for advanced solid tumours, and no patients were disengaged from the study due to adverse events. In the second phase of the experiment, pictilisib in combination with carboplatin-paclitaxel and bevacizumab was studied in individuals who had non-squamous non-small cell lung cancer (NSCLC), and tumour reduction has been witnessed in 6 patients. Therefore, pictilisib demonstrated preliminary antitumor activity as a single agent as well as in combination treatment in this study^57^. For pictilisib, multiple studies are still needed to explore the appropriate combination therapy for different patient groups.

#### Sonolisib/PX-866

Sonolisib is an oral PI3K inhibitor that can covalently bind to the ATP-binding site of the catalytic subunit of kinase p110 to effectively inhibit class IA PI3K^58^. As a wortmannin derivative, it is more stable than wortmannin but exhibits lower liver toxicity^58^. At present, clinical research is devoted to the efficacy of sonolisib in combination with other drugs, aiming to improve its antitumor activity, but the results are not satisfactory. In several phase II randomised trials, sonolisib failed to improve progression-free survival (PFS) as well as overall survival (OS) when combined with cetuximab or docetaxel in different types of cancer. As a result, the therapeutic potential of these combination therapies is restricted^59–62^. When raloxifene and sonolisib are combined, the expansion of breast cancer cells is significantly reduced, proving that the combination therapy has certain potential for patients with breast cancer^63^. In recent studies, sonolisib has been shown to inhibit temozolomide-induced autophagy and promote apoptosis in GBM cells, thereby enhancing antitumor efficacy. This provides the rationale for sequential therapy with temozolomide and sonolisib^64^. At present, the research progress of sonolisib is less published, and the use of sonolisib needs to be further studied.

#### Pilaralisib/XL147/SAR245408

Pilaralisib is a reversible, potent class I PI3K inhibitor. By inhibiting the formation of PIP3, pilaralisib inhibits the phosphorylation of downstream targets and is therefore useful in various cancer indications^65^. Both the capsule and tablet formulations of pilaralisib have been demonstrated to have anticancer effects^66^^,^^67^. In a clinical trial, patients with nonsteroidal aromatase inhibitor-refractory, recurrent, or metastatic HR^+^/HER2^−^ breast cancer were given pilaralisib or voxtalisib in combination with letrozole tablets. The PFS rates at 6 months in the two groups were 17% and 8%, respectively, indicating that the pilaralisib group had a better pharmacodynamic effect^68^. When pilaralisib was used in patients with chronic lymphocytic leukaemia (CLL) or lymphoma, PFS > 6 months was observed in 56.0% of patients^69^. Preliminary clinical activity has been demonstrated in studies, indicating that further development is warranted. A phase II experiment of pilaralisib in endometrial carcinoma patients observed an ORR of 6.0% and a PFS of more than 6 months in 11.9% of patients. As a result, pilaralisib has restricted clinical activity^70^. Moreover, the combination of paclitaxel and carboplatin with pilaralisib did not enhance the antitumor efficacy of patients with solid tumors^71^. Therefore, further clinical studies are needed to evaluate effective combination strategies with other inhibitors. Interestingly, in *in vitro* settings, it was found that pilaralisib-treated tumour cells were rapidly damaged by blue-wavelength (430 nm) light irradiation, and the degree of cell damage/death had to be dependent on the dose of pilaralisib and the light power of the irradiation. Pilaralisib accumulates in tumour cells when exposed to blue light and can be photosensitised by blue light to generate reactive oxygen species (ROS), causing tumour cell destruction. This opens up new avenues for using anticancer medicines in photodynamic therapy^72^.

### Isoform-specific inhibitors

**Figure 3.**
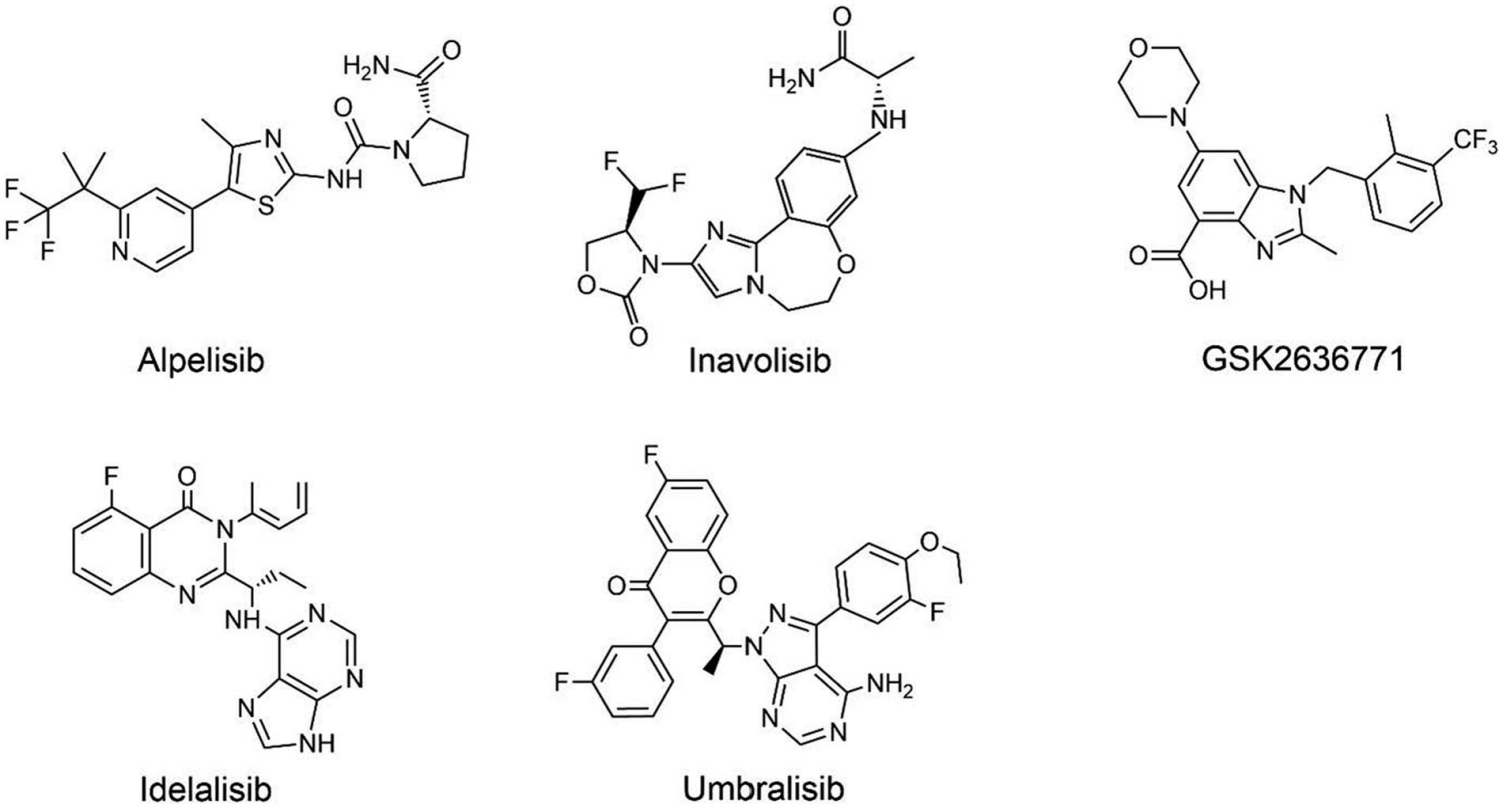
Structures of PI3K isoform-specific inhibitors.

Isoform-specific inhibitors are more popular because of their ability to selectively restrict different PI3K isoforms with less off-target toxicity. At present, several inhibitors have entered clinical studies, among which alpelisib, idelalisib, and umbralisib have been approved. The structures of PI3K isoform-specific inhibitors are shown in Figure 3.

#### Alpelisib/BYL719

Alpelisib belongs to the 2-aminothiazole family and is a PI3K inhibitor that can selectively inhibit the p110α subtype, which has great potential in breast cancer treatment^73^. A combination of alpelisib and fulvestrant was tested in the phase III trial to treat women with HR^+^/HER2^-^ breast cancer. Patients in the *PIK3CA* mutation cohort who received alpelisib had significantly longer median PFS in comparison to placebo (11 months vs. 5.7 months, respectively), and median OS was 39.3 months vs. 31.4 months, respectively. In the cohort without *PIK3CA*-mutated cancers, no clinically relevant treatment benefit was observed with alpelisib plus fulvestrant^74^. Hyperglycaemia, rash, and diarrhoea are the most frequently occurring side effects of alpelisib treatment. Because of the drug’s short half-life, the toxicity is reversible when the drug is interrupted or it can be controlled with early intervention and supportive therapy^75^. Given the findings of this phase III trial, the FDA approved alpelisib plus fulvestrant in May 2019 for patients suffering from HR^+^/HER2^-^ advanced breast cancer with *PIK3CA* mutations^76^. A phase 1b clinical study recently found that alpelisib plus olaparib was tolerable in patients suffering from advanced triple-negative breast cancer (TNBC), with 59% of patients achieving disease control and an ORR of 18%^77^. Besides, given the bidirectional crosstalk of oestrogen receptor (ER) and HER2 signalling, and the role of PI3K in mediating resistance to anti-HER2 drugs and endocrine therapy, it is speculated that simultaneous inhibition of PI3K, HER2, and ER pathways may be a reasonable therapeutic modality. A phase III randomised trial was therefore planned to evaluate alpelisib plus trastuzumab with or without fulvestrant in patients with *PIK3CA*-mutated HER2^+^ advanced breast cancer^78^.

#### Inavolisib/GDC-0077

Inavolisib is an innovative PI3K inhibitor with over 300-fold selectivity for PI3Kα isoforms over other class I PI3K isoforms, thus showing remarkable selectivity for PI3Kα isoforms^79^. *PIK3CA*-encoded p110α mediates most metabolic responses to insulin, so targeting it disrupts glucose metabolism across multiple tissues^80^. Insulin signalling inhibition, for example, promotes hepatic glycogenolysis while inhibiting glucose uptake in skeletal muscle and adipose tissue, leading to transitory hyperglycaemia following PI3K inhibition. The pancreas can release compensatory insulin (insulin feedback) to restore the body to normal glucose homeostasis, however this insulin feedback may reactivate PI3K/AKT/mTOR signalling in tumours, thereby affecting the effect of targeted therapy^80^. The development of PI3Kα mutant-selective inhibitors to avoid wild-type PI3Kα enzymes is a potential solution to this problem. As a PI3Kα selective inhibitor and mutant PI3Kα degrader, inavolisib can degrade mutant p110α protein without significantly altering wild-type p110α protein, so it can effectively improve the efficacy of *PIK3CA* mutant tumour patients^81^. Inavolisib exhibits strong efficacy in breast cancer patients, and several clinical studies have already been performed in combination with other drugs. Inavolisib, in conjunction with palbociclib and fulvestrant, demonstrated preliminary antitumor activity and manageable safety in patients with PIK3CA-mutated breast cancer^82^. Several clinical trials are currently underway for the treatment of breast cancer patients.

#### GSK2636771

GSK2636771 is a potent and orally selective PI3Kβ inhibitor that reduces off-target effects compared to pan-PI3K inhibitors^83^. The PI3Kβ isoform is an important lipid kinase that regulates the PI3K signalling pathway and the survival of *PTEN*-deficient tumour cells, so PI3Kβ inhibitors have a role in *PTEN*-deficient cancers and are expected to improve clinical efficacy through drug combination^84^. GSK2636771 demonstrated favourable clinical benefit and a manageable safety profile in *PTEN*-deficient and/or *PIK3CB*-abnormal cancer patients in a first-in-human clinical study^83^. β-subtype-specific inhibitors can significantly inhibit the migration and invasion of prostate cancer cells at lower concentrations, showing strong *in vitro* anti-tumour metastatic activity^85^. Thus, in a phase, I study of GSK2636771 in combination with the androgen receptor antagonist enzalutamide in patients with metastatic castration-resistant prostate cancer (mCRPC), a 12-week non-progressive disease incidence of 50% was observed^86^. Additionally, the clinical benefit rate of GSK2636771 in combination with pembrolizumab in patients with *PTEN*-deficient melanoma or other advanced solid tumours was 52%^87^. Although the combination of GSK2636771 is well tolerated, the antitumor activity shown is still limited.

#### Idelalisib

Idelalisib, a PI3Kδ inhibitor for continuous oral use, was approved by the FDA in 2014 for the treatment of relapsed CLL, relapsed follicular B cell NHL, and relapsed small lymphocytic lymphoma (SLL)^88^. The combination of idelalisib and rituximab demonstrates a significant advantage in patients with relapsed CLL, with ORRs of 81% in the idelalisib group and 13% in the control groups, respectively^89^. The trial was terminated early due to the significant efficacy of the idelalisib arm, and an extension study was initiated, in which patients in the first two arms could participate in an extension study to receive idelalisib monotherapy. Results show that idelalisib significantly improves PFS and OS in patients with relapsed CLL compared with rituximab alone^90^. Given that targeting PI3Kδ leads to impairment of the T cell immune response, there have been some adverse events such as PJP, cytomegalovirus (CMV)-related death, and respiratory disease caused by infection^91^. In the extension study described above, 4 patients (3.6%) in the idelalisib group and 1 patient in the control group (0.9%) developed PJP infections. However, none of these five patients received anti-PJP prophylaxis, so the trial reinforces the PJP prophylaxis recommendation for idelalisib-treated patients^90^. Serious pulmonary complications including PJP infection and CMV reactivation may occur when idelalisib is used in combination with bendamustine. Therefore, PJP prevention and CMV surveillance are recommended^92^. Idelalisib frequently causes immune-mediated hepatotoxicity in first-line therapy, and younger subjects and those with immunoglobulin heavy chain (IGHV)-mutated disease are more likely to experience early hepatotoxicity^93^. Idelalisib treatment is also accompanied by side effects such as severe diarrhoea, colitis, pneumonia, and intestinal perforation, making the clinical discontinuation rate as high as 50%^94^. Several relevant clinical trials have been conducted, but the use of idelalisib is usually restricted because of its severe toxicity. In addition, Gilead announced the termination of the follow-up development plan and withdrew the indications of idelalisib for the treatment of FL and SLL in 2022, which is basically equivalent to withdrawing from the market.

#### Umbralisib

Umbralisib is an oral dual inhibitor of PI3Kδ and casein kinase 1ε (CK1ε) with improved selectivity for the PI3K isoform, while inhibiting CK1 signalling reduces the autoimmune toxicity associated with this class of drugs^95^. From an analysis of comprehensive toxicity data, 13.7% of patients withdrew treatment for adverse effects caused by umbralisib treatment, while the rates of discontinuation owing to adverse effects in clinical studies of idelalisib, copanlisib, and duvelisib were 20.0%, 25%, and 52.0%, respectively. In contrast, umbralisib showed better tolerability and therapeutic potential^96^. In 2021, umbralisib was licenced in the United States for the treatment of adults with relapsed or refractory MZL and FL^97^. Multiple clinical studies have explored umbralisib-related combination therapy effects. In a phase III trial, patients with relapsed/refractory CLL were given umbralisib plus ublituximab (U2) and obinuzumab plus chlorambucil (O + Chl). Both the U2 group and the O + Chl group significantly prolonged PFS, with ORRs of 83.3% and 68.7% respectively, and 34 (16.5%) and 16 (7.6%) patients discontinued treatment due to adverse effects in the two groups respectively^98^. Therefore, it can be stated that the combination therapy has a high degree of feasibility. However, the efficacy of umbralisib combination therapy is still being explored, hoping to bring new breakthroughs to the treatment of patients with hematological malignancies.

### Dual PI3K/mTOR or dual PI3K inhibitors

**Figure 4.**
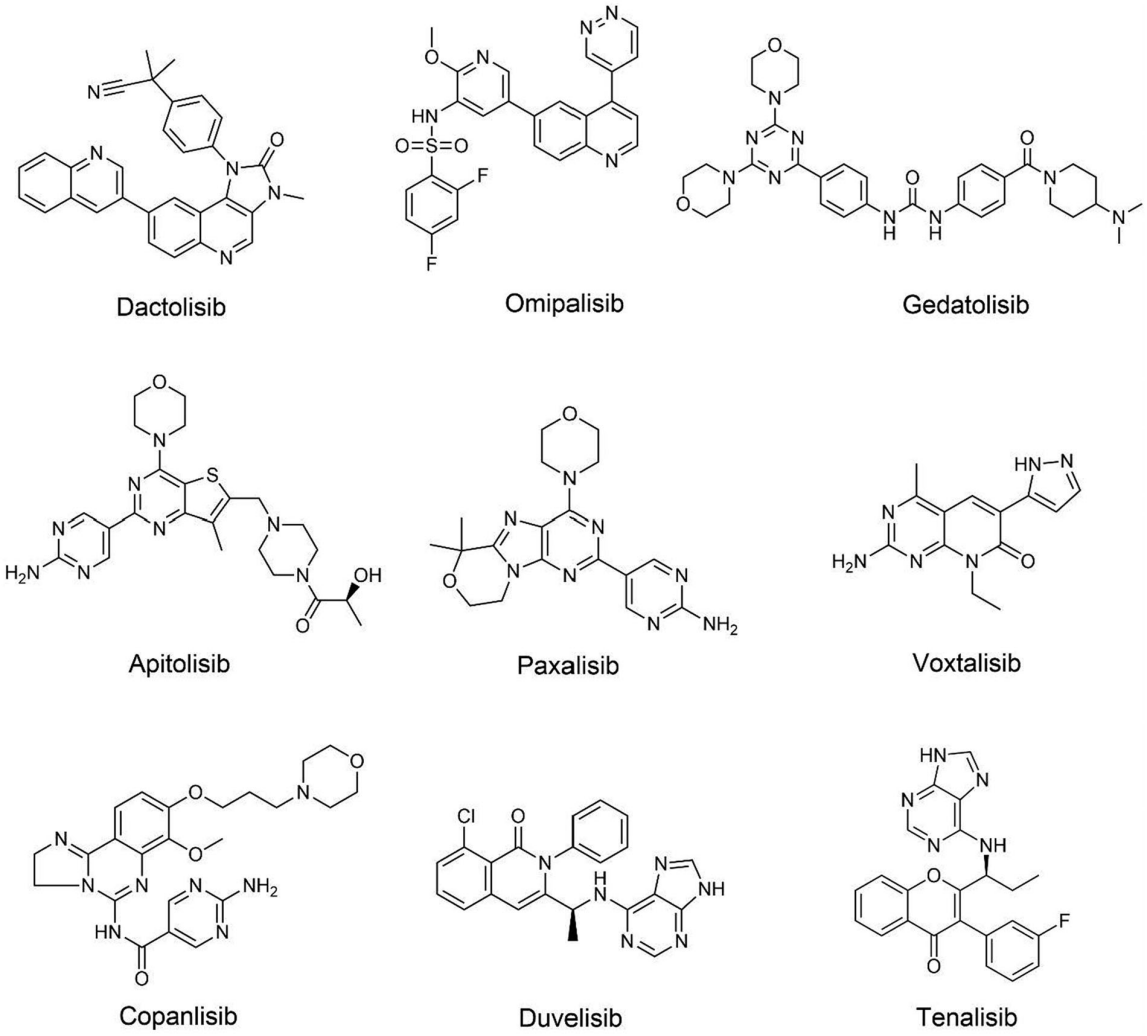
Structures of dual PI3K/mTOR or dual PI3K inhibitors.

PI3K/mTOR or dual PI3K inhibitors can more completely inhibit the PI3K/AKT/mTOR signalling pathway and the occurrence of negative feedback regulation, thus possessing a wider range of activities^42^^,^^99^. On the other hand, dual targeting will result in more unpredicted clinically relevant negative consequences and adverse reactions, making their innovation more complex and difficult. Therefore, the development of a high-efficiency and low-toxic dual inhibitor has become the research and development goal of such drugs. The structures of dual PI3K/mTOR or dual PI3K inhibitors are shown in Figure 4.

#### Dactolisib/BEZ-235/NVP-BEZ235

Dactolisib, an imidazoquinoline derivative, is the first dual PI3K/mTOR inhibitor to enter clinical trials with IC_50_s of 4, 5, 7, 75, and 20.7 nmol/L for p110α/γ/δ/β and mTOR, respectively^42^. Compared with first-generation mTOR inhibitors, dactolisib can simultaneously inhibit mTORC1 and mTORC2, thereby avoiding the activation of feedback regulatory pathways^100^. In a phase II study of treatment-naïve patients with advanced pancreatic neuroendocrine tumours, the median PFS was 8.2 months with dactolisib compared with 10.8 months with everolimus. Dactolisib did not show higher efficacy than everolimus due to the poor tolerability of dactolisib^101^. Similarly, tolerability issues have limited further clinical studies of dactolisib in renal cell carcinoma as well as prostate cancer^102^^,^^103^. Dactolisib is highly hydrophobic, so high-dose oral administration is often used in clinical trials. Due to the low bioavailability and high toxicity, the relevant clinical trial results are not satisfactory. Irreversible electroporation (IRE) is an emerging minimally invasive tumour ablation technique. When IRE is combined with an intratumorally injected liposomal formulation of dactolisib, IRE is able to release dactolisib from its liposomal encapsulation, thereby increasing its solubility in aqueous solutions^104^. This combined approach has significant antitumor effects and efficacy in limiting cancer cell proliferation, and is a promising combination therapy for cancer treatment. Unfortunately, BEZ-235 has now been withdrawn from development.

#### Omipalisib/GSK2126458/GSK458

Omipalisib, an oral and reversible dual inhibitor of PI3K/mTOR discovered by GlaxoSmithKline, showed broad antitumor activity in preclinical studies^105^. In a first-in-human phase I study, omipalisib treatment triggered durable objective responses in multiple tumour types, including endometrial, oropharyngeal, renal cell, and bladder cancer^105^. Only one patient achieved a partial response when the mitogen-activated protein kinase (MEK) inhibitor trametinib was combined with omipalisib, instead 42% of patients had dose interruptions due to adverse events^106^. Overlapping side effects are often seen in combination therapy, so omipalisib plus trametinib showed poor tolerance and poor clinical efficacy. Importantly, recent studies have shown that omipalisib can inhibit non-homologous end joining (NHEJ) and sensitise cancer cells to radiotherapy and chemotherapy, thus being a potential inhibitor of DNA double-strand break repair^107^. Omipalisib has shown limited efficacy in cancer treatment. However, PI3K and mTOR are involved in the pathogenesis of idiopathic pulmonary fibrosis (IPF). It has been shown that omipalisib was well tolerated by subjects with IPF, supporting further evaluation of omipalisib as a novel therapy for IPF^108^.

#### Gedatolisib/PF-05212384/PKI-587

Gedatolisib is an intravenous PI3Kα, PI3Kγ, and mTOR inhibitor developed by Pfizer. Gedatolisib can inhibit the activation of the PI3K/mTOR signalling pathway and induce G_0_/G_1_ cell cycle arrest, which has great potential for tumour therapy^109^. Two PI3K/mTOR inhibitors, PF-04691502 and gedatolisib, were administered in combination with irinotecan or PD-0325901 in a phase I study. Both combinations of PF-04691502 were discontinued early because of poor tolerance, and no maximum tolerated dose (MTD) for either group was established. The ORRs of gedatolisib combined with irinotecan or PD-0325901 were 4.7% and 11.1%, respectively, and preliminary antitumor activity was observed. Tolerability remains a non-negligible issue, however, and both groups require frequent dose delays and dose reductions to mitigate the toxicity associated with combination therapy^110^. In the phase II trial, PF-04691502 was compared with gedatolisib in subjects with recurrent endometrial carcinoma. Because of intolerable toxic effects, two PF-04691502 research groups were terminated soon. The clinical benefit response rates for the gedatolisib groups were 53% and 26%, respectively^111^. At present, in a phase I study in cancer patients, gedatolisib was combined with paclitaxel and carboplatin, the ORR is 65%, and the ORR of clear cell ovarian cancer is 80%, showing good tolerability and preliminary efficacy. A follow-up trial is therefore planned^112^.

#### Apitolisib/GDC-0980

Apitolisib, a novel oral PI3K/mTOR inhibitor developed by Genentech, has shown anti-tumour activity in breast, prostate, endometrial, and other cancers by blocking the cell cycle and thereby inducing apoptosis^113^. The phase I study demonstrated modest antitumor activity in patients with PIK3CA-mutated head and neck cancer, peritoneal mesothelioma, and pleural mesothelioma^114^. Furthermore, the most prevalent apitolisib-related toxicities were hyperglycaemia, pneumonia, rash, liver dysfunction, and diarrhoea^114^. Apitolisib and everolimus were assessed in a phase II study on people with renal cell carcinoma (mRCC). The results showed that apitolisib was not as effective as everolimus, with PFS of 3.7 months and 6.1 months, and ORR of 7.1% and 11.6%, respectively^115^. Apitolisib monotherapy is often inefficient, and its combination therapy with chemotherapeutics should be further explored. Studies have shown that apitolisib and the MEK inhibitor refametinib can exert an anti-proliferative effect on hypopharyngeal squamous cell carcinoma cell lines^116^. Apitolisib in combination with gemcitabine and/or cisplatin can improve chemotherapy sensitivity and antitumor activity in advanced cholangiocarcinoma^117^. In addition, recent studies have shown that apitolisib can inhibit the proliferation of human GBM cells, thus apitolisib is expected to be further evaluated in clinical trials aimed at GBM^118^. Therefore, apitolisib is a promising candidate for cancer treatment.

#### Paxalisib/GDC-0084

Paxalisib is an oral and selective PI3K/mTOR inhibitor that permeates the blood-brain barrier (BBB). A previously designed potent and BBB-penetrating PI3K inhibitor showed poor stability, and the purine-based inhibitor paxalisib was finally identified through continuous design optimization^119^. Paxalisib is a drug specifically designed to treat brain cancer. Paxalisib has been discovered in the latest research to dramatically reduce cell survival in *PIK3CA*-mutated breast cancer brain metastatic cell lines^120^. Given that paxalisib has shown some activity in improving intracranial tumours, further clinical trials are scheduled to test its effectiveness in treating brain metastases. In the phase I study in patients with high-grade glioma, paxalisib efficiently crossed the BBB, and nineteen patients (40%) achieved stable disease. Therefore, paxalisib has obvious potential to treat brain cancer^121^. According to interim results from a phase II study, the MTD for paxalisib in patients with newly diagnosed GBM was 60 mg/d. When all patients were included in the analysis, the PFS was 8.5 months, indicating a promising efficacy signal^122^. To achieve the maximum efficacy of paxalisib, clinical studies in combination with other medications are still needed to meet the urgent need for the management of brain tumours.

#### Voxtalisib/XL765/SAR245409

Voxtalisib has the strongest effect on p110γ, while also inhibiting mTOR. There have been several human studies of voxtalisib with other chemotherapeutic agents. The phase I/II study compared pilaralisib and voxtalisib plus letrozole in breast cancer patients. PFS rates at 6 months were 17% in the pilarisib group and 8% in the voxtalisib group, and in the combination therapy cohort both demonstrated limited clinical activity^68^. When voxtalisib was coupled with temozolomide in patients diagnosed with high-grade glioma, the partial response was estimated to be 4%, of which 68% had stable disease^123^. When voxtalisib was combined with the MEK inhibitor pimatinib in cancer patients, 51 patients (46%) achieved stable disease^124^. In recent years, voxtalisib monotherapy demonstrated promising efficacy in FL patients in a phase II trial, with an ORR of 41.3% and a complete response rate of 10.9%. However, in patients with mantle cell lymphoma, diffuse large B cell lymphoma or CLL/SLL, efficacy is limited^125^. Currently, the efficacy of voxtalisib in different tumour settings is being further explored.

#### Copanlisib/BAY 80–6946

Copanlisib, an intermittent intravenous inhibitor, inhibits four PI3K isoforms and significantly inhibits the activity of PI3Kα/δ isoforms^126^. The FDA has authorised copanlisib monotherapy for the treatment of adult individuals with relapsed and refractory follicular lymphoma (FL), as well as for pre-treatment breakthrough designation for patients with marginal zone lymphoma (MZL)^127^. FL is refractory, and most patients usually have disease recurrence^128^. At present, the optimisation scheme for FL treatment is constantly advancing.

Copanlisib exhibits significant efficacy and controllable safety in the therapy of indolent non-Hodgkin lymphoma (iNHL). CHRONOS-1 (phase II trial) for iNHL included parts A and B, and the objective response rates (ORR) were 43.8% and 59.2%, respectively^129^. According to the findings of phase III clinical study CHRONOS-3, the PI3K inhibitor copanlisib + rituximab group diminished the probability of disease development/death by 48% when contrasted to the placebo group, proving that copanlisib combined with rituximab has good efficacy in the treatment of relapsed iNHL patients (including MZL)^130^. The phase III clinical trial CHRONOS-4 was designed to compare copanlisib combined with rituximab plus bendamustine (RB) with copanlisib combined with rituximab plus cyclophosphamide, doxorubicin, vincristine, and prednisone (R-CHOP) in patients with relapsed indolent B cell lymphoma. The ORRs of the copanlisib plus RB and R-CHOP groups were 90% and 100%, respectively. Due to the small number of patients, further trials are still needed to confirm these clinical data^131^. The most common adverse reactions to copanlisib treatment are temporary hyperglycaemia/hypertension, which can recover to baseline levels after reaching their peak^132^^,^^133^. There is a significant acute liver injury during copanlisib therapy, therefore it is recommended that patients receiving copanlisib are monitored for liver functions^134^. The most prevalent infection in treated patients is pneumonia, for which *Pneumocystis jirovecii* pneumonia (PJP) prophylaxis is recommended^134^. At present, the combination of copanlisib and afatinib for the treatment of head and neck squamous cell carcinoma (HNSCC) can markedly restrict the growth and survival of tumour cells *in vitro*, which would be a potential method to replace cisplatin-resistant therapy^135^. Furthermore, *PIK3CA* is the most frequently mutated oncogene, and copanlisib was also tested in patients with *PIK3CA* mutations in the NCI-MATCH (EAY131) trial. The trial revealed encouraging therapeutic efficacy in refractory tumours with *PIK3CA* mutations, with an ORR of 16%^136^. copanlisib shows greater safety and clinical potential for cancer treatment than other similar drugs, and further in-depth study of its clinical effects is needed.

#### Duvelisib

Duvelisib targets vital tumorigenic routes by inhibiting the γ and δ isoforms of PI3K, which is not only directly toxic to tumour cells but also abrogates pro-survival cytokine and chemokine signalling in the tumour microenvironment^137^. In the phase I trial, duvelisib demonstrated favourable efficacy in patients suffering from advanced malignant tumours, including those with high-risk biologics. The ORR was 46% in patients with 17p deletion or *TP53* mutation and 51% in patients with unmutated IGHV^138^. The ORR in the phase II DYNAMO trial of duvelisib monotherapy in patients with iNHL was 47.3%, with a median PFS of 9.5 months. As a result, monotherapy appears to have an encouraging clinical benefit^139^. In the phase III DUO trial, duvelisib was contrasted to ofatumumab in patients with CLL/SLL. The results showed that the PFS was 13.3 months and 9.9 months respectively, and the ORR was 74% and 45%, respectively^140^. Based on the significant efficacy of duvelisib in this trial, duvelisib received the first global approval in the United States for the treatment of CLL/SLL adult patients^141^. When duvelisib was combined with other drugs in patients with NHL and CLL, the ORR in group 1 (duvelisib plus rituximab) was 78.3%, and the ORR in group 2 (duvelisib plus bendamustine and rituximab) was 62.5%^142^. Combination therapy showed no superimposed toxicity, so concomitant use is safe and feasible. Duvelisib is very similar to idelalisib, and expanded use will be limited by toxicities that fall into two broad categories: immune-mediated and infectious^143^. Measures should be taken to monitor, for example, in patients receiving duvelisib, CMV, and PJP prophylaxis is strongly suggested to lower the risk of some possibly deadly toxicities^144^.

#### Tenalisib (RP6530)

Tenalisib is a potent dual PI3K δ/γ inhibitor with _IC50s_ of 25 nM and 33 nM, respectively^145^. Tenalisib, as a single drug, showed significant clinical activity in the treatment of T cell lymphoma (TCL) patients, with a total remission rate of 45.7%^146^. Subsequently, the efficacy evaluation of tenalisib combined with romidepsin in the treatment of peripheral TCL and skin TCL was conducted, and the results showed that the ORR was 75% and 53.3%, respectively^147^. Therefore, both monotherapy and combination therapy with tenalisib have shown high safety and excellent clinical activity. At present, Rhizon Pharmaceutical Company has announced that tenalisib has been approved by the FDA as a fast-track and Orphan drug for the treatment of PTCL^145^. Besides, tenalisib also carried out a phase II study on metastatic breast cancer (NCT05021900)^148^. Tenalisib is still being studied clinically for solid tumours and hematological malignancies.

## Phosphoinositide 3-kinase-related protein kinases (PIKK)

PIKKs are a group of atypical serine/threonine kinases with conserved domain structures. From N-terminal to C-terminal, these are the FRAP-ATM-TRRAP (FAT) domain, the kinase domain (KD), the PIKK-regulatory domain (PRD), and the FAT-C-terminal (FATC) domain, with KD having significant sequence homology with PI3K^149^. There are currently six PIKK members, including ATM, ATR, DNA-PK, mTOR, SMG-1, and TRAAP. Members closely related to DNA damage repair (ATM, ATR, and DNA-PK) can detect DNA damage, activate cell cycle checkpoints, and initiate DNA repair signalling cascades^150^. mTOR is capable of regulating nutrient-dependent signalling, SMG-1 mainly controls nonsense-mediated mRNA decay, and TRRAP is capable of regulating transcription but lacks kinase activity^151–153^. Based on the correlation between the above proteins and cancer and the breadth of research, we mainly introduce the PIKK members ATM, ATR, and DNA-PK related to DRR, as well as the key target mTOR of the PI3K signalling pathway.

**Figure 5.**
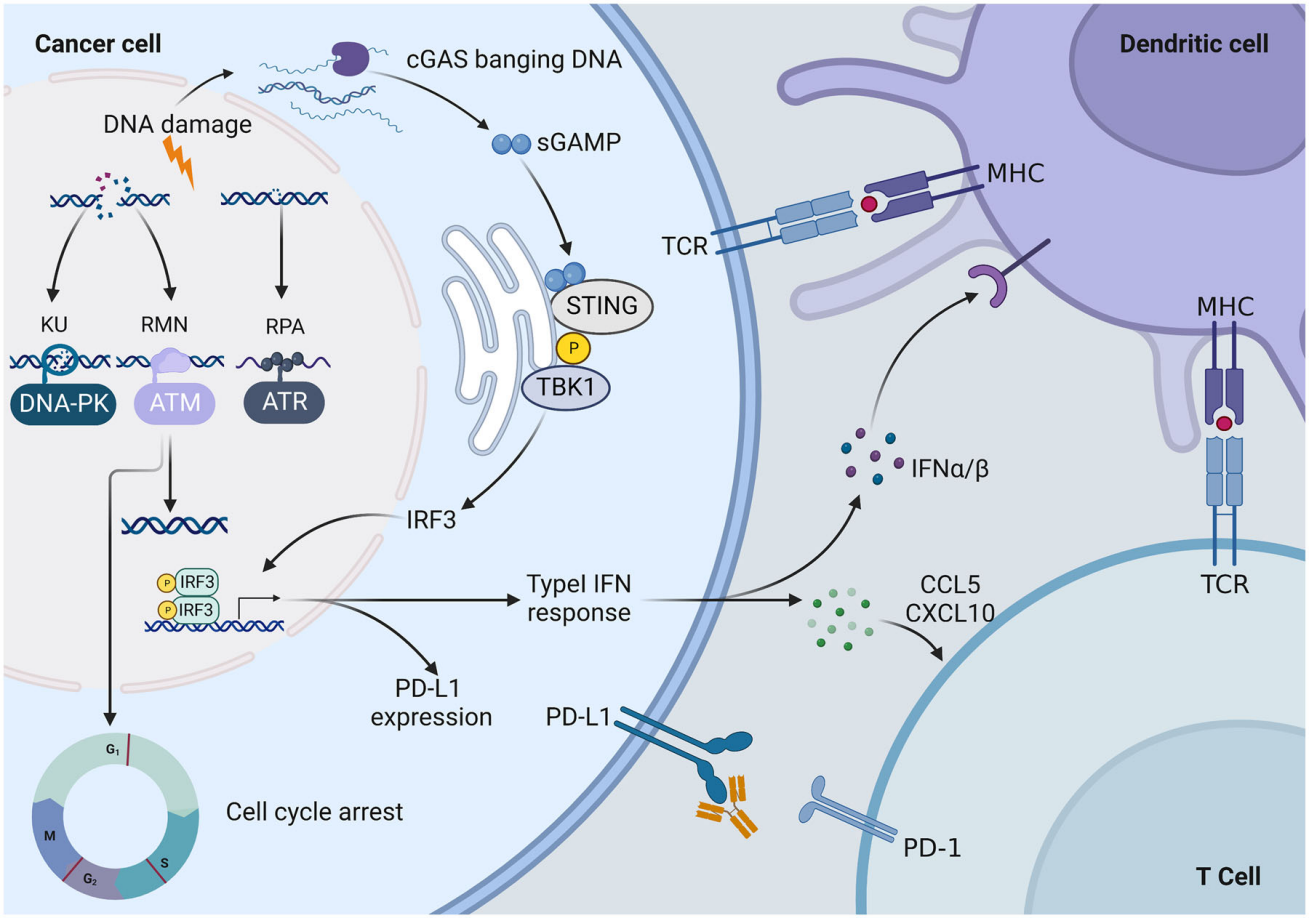
PIKK mediated DNA damage response (DDR) inactivation regulates the immune response. PIKK members ATM, ATR, and DNA-PK have the ability to promote cell cycle arrest and DNA repair. DNA-PK, *via* the Ku heterodimer, binds to double-strand broken DNA ends, promoting non-homologous end-joining (NHEJ). The MRN complex recruits ATM to single-strand broken DNA, which is then repaired *via* homologous recombination (HR). In response to HR, ATR is recruited to single-stranded DNA wrapped by replicating protein A (RPA) for DNA repair. DNA damage response (DDR) gene inactivation leads to the accumulation of chromosomal fragments in the cytoplasm, which activates the cyclic GMP-AMP synthase-interferon gene stimulator (cGAS-STING) pathway. STING activates interferon regulatory factor 3 (IRF3) transcription by binding to and activating tank-binding kinase 1 (TBK1). The type I IFN response is upregulated by the chemokine C-X-C motif, chemokine ligand 10 (CXCL10) and chemokine receptor 5 (CCL5), thereby promoting the infiltration of effector T cells into tumours. In addition, the expression of programmed cell death 1 ligand 1 (PD-L1) was upregulated. Secreted IFN-α and IFN-β promoted the antigen-presenting ability of dendritic cells.

DNA is susceptible to damage by intrinsic and extrinsic toxic effects, including ROS, ultraviolet radiation, and ionising radiation, so the body develops a complex and organised DDR mechanism to preserve the genome’s integrity^154^. Double-strand breaks (DSBs) are one of the most virulent types of DNA lesions, and their repair is accomplished by two distinct mechanisms: homologous recombination (HR) and NHEJ^155^. ATM is mainly engaged in HR repair and recruited to DNA ends, and it is activated by binding to the MRE11-RAD50-NBS1 complex^156^. DNA-PK is a complex made up of a catalytic subunit (DNA-PKcs) and a Ku70/80 heterodimer (Ku70/80), and full activation of DNA-PKcs occurs only in the presence of DNA and Ku70/80^157^. Single-strand breaks (SSBs) are repaired by ATR through homologous recombination. ATR is recruited to single-stranded DNA coated by replication protein A (RPA) by binding to ATR interacting protein (ATRIP), which is then activated by DNA topoisomerase 2-binding protein 1 (TOPBP1)^158^^,^^159^. ATM, ATR, and DNA-PK are situated at the upper end of the signalling pathway, activating multiple downstream targets. ATR and ATM kinases, respectively, regulate the activity of checkpoint kinase 1 (Chk1) and checkpoint kinase 2 (Chk2)^160^. Subsequently, Chk1 and Chk2 can regulate certain substrates associated with cell cycle arrest, such as cell division cyclin25 family phosphatases, resulting in cell cycle stagnation at G2/M and S, respectively^160^. DNA-PK phosphorylates different residues on the tumour suppressor gene p53, thereby inhibiting the ubiquitination and degradation of p53 by murine double microgene 2 (MDM2), leading to G1 cell cycle stagnation^161^. In summary, the ATM, ATR, and DNA-PK signalling pathways are interconnected and intertwined, producing a complex signalling network that can promote DNA damage repair, trigger cell cycle checkpoints, and cause cellular senescence or apoptosis in cells^5^. Since the DNA repair mechanism within cancer cells can lead to resistance to DNA-damaging anti-cancer therapies, blocking DNA repair in cancer cells has the potential to boost sensitivity to chemotherapy and radiotherapy^162^^,^^163^. Moreover, there is a close relationship between the immune response and DNA repair pathways. The replication stress response (RSR) is defined as the activation of the DDR pathway and the inhibition of cell cycle progression^164^. Small-molecule inhibitors of RSR components such as ATM, ATR, and DNA-PK can enhance the instability of the tumour genome to stimulate innate immune responses^165–167^. DDR gene inactivation results in the accumulation of chromosome fragments in the cytoplasm, which can be bound by cytoplasmic cyclic GMP-AMP synthase (cGAS)^168^. DNA binding causes conformational changes in cGAS that facilitate the synthesis of cGAMP, which activates the interferon gene stimulator (STING) and promotes its localisation to the Golgi apparatus. Subsequently, Tank-bound kinase 1 (TBK1) was recruited to phosphorylate interferon regulatory factor 3 (IRF3), and the activated IRF3 entered the nucleus to promote the transcription of type-I interferon (IFN) genes^168^. Type I IFN signalling promotes the activation of dendritic cells, allowing them to present tumour antigens to CD8 T cells^169^. Type I IFN response up-regulates chemokine C-X-C motif chemokine ligand 10 (CXCL10) and chemokine ligand 5 (CCL5), which can effectively promote the infiltration of effector T cells into the tumor^164^. However, the GAS-STING pathway can result in increased expression of programmed cell death ligand 1 (PDL-1)^170^ (Figure 5). The combination of DNA damage agents and immunotherapy seems to be a promising treatment, but its effectiveness still needs further study.

mTOR can form two structurally distinct protein complexes, namely mTORC1 and mTORC2. mTORC1 is a rapamycin-sensitive protein kinase composed of three core components, namely mTOR, the regulatory-associated protein of mTOR (Raptor), and mammalian lethal with Sec13 protein 8 (mLST8, also known as GβL)^171^^,^^172^. In addition, it also contains two non-core components, namely proline-rich AKT1 substrate 1 (RAPTOR) and the DEP domain-containing mTOR-interacting protein (DEPTOR)^173^^,^^174^. Various growth factors can activate mTORC1 by stimulating signalling pathways involving PI3K and its downstream effector AKT. In this process, activated AKT mediates TSC2 phosphorylation and inhibits the TSC complex, thereby preventing negative regulation of the Ras homologue enriched in the brain (Rheb) by the complex, allowing for Rheb enrichment and activation of rapamycin-sensitive mTORC1^175^. Ribosomal protein S6 kinase 1 protein (S6K1) and eukaryotic initiation factor 4E-binding protein (4E-BP1) are the major phosphorylation targets of mTORC1, both of which are involved in translation and protein synthesis^176^. In addition, S6K1 can directly phosphorylate insulin receptor substrate 1 (IRS-1) as part of the negative feedback loop, thereby reducing insulin-based PI3K activation^177^. mTORC2 also includes mTOR and mLST8. The difference is that there is no Raptor in mTORC2, but rather a rapamycin-insensitive companion of mTOR (Rictor) instead^178^^,^^179^. In addition to its core components, mTORC2 also contains DEPTOR as well as regulatory subunits mSin1 and Protor1/2^174^^,^^180^^,^^181^. mTORC2 can phosphorylate AKT to regulate signalling pathways, control cytoskeleton rearrangement, and regulate glucose metabolism, as well as participate in cell growth, apoptosis, and cell cycle control^182^. Collectively, mTOR is able to participate in intracellular and extracellular signalling, regulating multiple downstream pathways involved in a range of pathophysiological processes, such as cancer, metabolic disorders, neurodegeneration, and aging^183^.

## PIKK inhibitors

Members of the PIKK family engage in a wide range of physiological functions, and their functional abnormalities can lead to various diseases, including cancer. Therefore, targeted inhibition of PIKK family members has broad prospects for treating cancer. Due to the similar kinase domains of PI3K and PIKK, the chemical inhibitors of the two family kinases seem to share similar structural motifs^184^. Currently, many studies on DNA damage repair and mTOR-related inhibitors are ongoing, and some drugs have entered clinical trials. However, only mTOR inhibitors have been approved.

### ATR, ATM, and DNA-PK inhibitors

**Figure 6.**
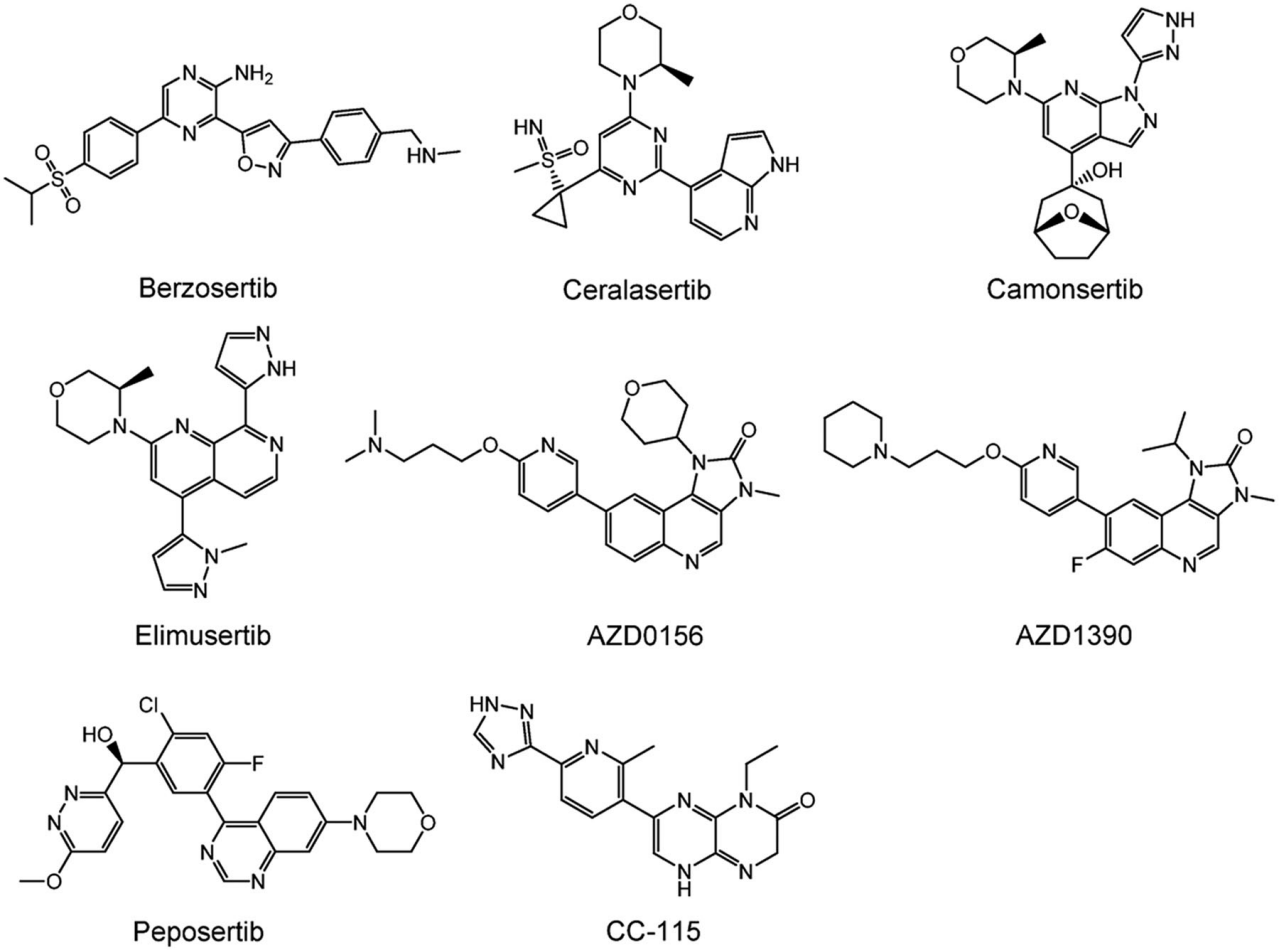
Structures of PIKK family members ATR, ATM, and DNA-PKcs inhibitors.

DDR is crucial for maintaining genomic stability in the body. However, inhibiting DNA repair in tumours can significantly improve the sensitivity of genotoxicity therapy (chemotherapy and radiotherapy)^162^^,^^163^. Moreover, the use of synthetic lethal relationships can selectively kill tumour cells with specific DDR pathway defects^20^. Therefore, DDR-related inhibitors have become cancer-specific therapeutic targets. DDR inhibitors are promising targets for breaking resistance to standard care treatments, with relatively mild and tolerable side effects. Currently, the anticancer therapy hotspots ATM, ATR, and DNA PK are members of the PIKK family, and several inhibitors are in preclinical and clinical development. However, no medications have yet received approval (Table 2). The structures of PIKK family members ATR, ATM, and DNA-PK inhibitors are shown in Figure 6.

**Table 2.** A summary of major PIKK inhibitors.

Inhibitor	Compound	Inventor	IC_50_ (nmol/L)	Phase	Clinical
ATR inhibitors	Berzosertib(VX-970)	Merck	ATR: 19	II-Ongoing	NCT04768296
	Ceralasertib(AZD6738)	AstraZeneca	ATR: 1	II-Ongoing	NCT03780608
	Camonsertib(RP-3500)	Repare Therapeutics	ATR: 0.33	I-Ongoing	NCT04497116
	Elimusertib(BAY-1895344)	Bayer	ATR: 7	I	NCT03188965
ATM inhibitors	AZD0156	AstraZeneca	ATM: 0.58	I-Ongoing	NCT02588105
	AZD1390	AstraZeneca	ATM: 0.78	I-Ongoing	NCT03423628
DNA-PK inhibitors	Peposertib(M3814)	Merck	DNA-PK: < 3	I	NCT03770689
	CC-115	Bristol-Myers Squibb	mTOR/DNA-PK:21/13	II-Ongoing	NCT02977780
mTOR inhibitors	nab-sirolimus(ABI-009)	Aadi Bioscience	——	Approved	
	Temsirolimus(CCI-779)	Wyeth	mTOR: 1.76	Approved	
	Everolimus(RAD-001)	Novartis	mTOR: 1.6–2.4	Approved	
	Ridaforolimus	Merck and Ariad	mTOR: 0.2	Terminated	
	Vistusertib(AZD2014)	AstraZeneca	mTOR: 2.8	I/II-Ongoing	NCT02730923
	Sapanisertib(TAK-228)	Takeda (Japan)	mTOR: 1	II	NCT02756364

#### Berzosertib/VX-970/VE-822/M6620

Berzosertib is a powerful ATR inhibitor administered intravenously and is currently being evaluated by Merck. To improve the targeted therapy of selective inhibitors, it is possible to target defective pathways in cancer cells, use synthetic lethal interactions to kill cancer cells or rely on synergy with other drugs to enhance anticancer effects^185^. In the phase I study, the berzosertib plus gemcitabine group had a partial response of 8.3% and a stable disease of 60.4%^186^. In the berzosertib plus gemcitabine plus cisplatin group, partial response (PR) was 14% and stable disease was 57%. Additionally, berzosertib was well tolerated only in combination with cisplatin-free gemcitabine, so the suggested phase II dose (RP2D) for this combination was established^186^. Following that, a phase II trial of berzosertib plus gemcitabine in high-grade serous ovarian cancer was conducted. Berzosertib combined with gemcitabine significantly prolonged PFS compared to gemcitabine alone (22.9 weeks vs. 14.7 weeks), and the combination therapy reduced the proportion of patients with severe adverse reactions (26% vs. 28%)^187^. Berzosertib has good BBB penetration in preclinical studies of GBM. Studies have shown that berzosertib exhibits a radiosensitizing effect in preclinical NSCLC brain metastases models, so a clinical trial of berzosertib combined with whole brain irradiation for patients with NSCLC brain metastases is currently underway^188^.

#### Ceralasertib/AZD6738

Ceralasertib is a potent and selective oral ATR inhibitor currently being evaluated by AstraZeneca. Ceralasertib elicits promising antitumor activity in preclinical studies, so multiple clinical trials were conducted to evaluate the effectiveness of ceralasertib in combination with various chemotherapeutic drugs. Ceralasertib plus carboplatin or paclitaxel demonstrated excellent anticancer potential in phase I studies in cancer patients, providing a recommended RP2D^189^^,^^190^. Besides, ceralasertib plus durvalumab showed promising clinical effects in patients with metastatic melanoma and advanced gastric cancer. The ORRs were 31.0% and 22.6% respectively, and the disease control rates were 63.3% and 58.1% respectively. Common adverse events were mainly haemorrhagic and could be managed with dose interruptions and reductions^191^^,^^192^. In contrast, the combination of ceralasertib and olaparib was less beneficial for treating genetically mutated ovarian cancer patients, with an ORR of 8.3%^193^. The phase II study of this medication plan was then carried out, and the results showed that 9 patients had stable disease. Active signalling is especially observed in *BRCA1*-related diseases. Since no objective response has been received, further evaluation of this combination is required^194^.

#### Camonsertib/RP-3500

Camonsertib is an oral ATR inhibitor that is currently undergoing clinical evaluation. In a clinical study related to camonsertib, it was shown that poly (adenosinediphosphate-ribose) polymerase (PARP) inhibitors not only block DNA break repair but also stabilise the PARP-DNA complex, thereby stopping the replication fork. Therefore, both effects can lead to the accumulation of DSB in DNA replication and its dependence on HR^195^. The combination of ATR and PARP showed excellent antitumor activity in preclinical models. However, this combination results in overlapping toxicities, including myelosuppression, that limit tolerance to the combination. Therefore, the researchers used a short-term intermittent dosing regimen when combining camonsertib with a PARP inhibitor, and the results showed that this method can maximise the efficacy and alleviate the occurrence of anaemia, which is a potential method for cancer treatment^195^. Based on the good preclinical anticancer effect, the phase I study related to camonsertib is currently underway.

#### Elimusertib/BAY-1895344

Elimusertib is a potent, orally available, and selective ATR inhibitor. In preclinical models, elimusertib monotherapy showed anti-tumour activity in prostate cancer, colorectal cancer, and mantle cell lymphoma models, with the most sensitive response in lymphoma^196^. Given the promising activity of elimusertib in preclinical testing, multiple clinical trials were conducted to evaluate its efficacy in humans. The MTD of the phase I clinical study was 40 mg. Among patients treated at the MTD or higher, the disease control rate was 69.2%, the ORR was 30.8%, and the median time to response was 78 d^197^. Elimusertib has shown preliminary clinical activity in first-in-human trials, and multiple trials are currently recruiting.

#### AZD0156

AZD0156 is an imidazo[4,5-c]quinolin-2-one core-based selective ATM inhibitor and the first ATM inhibitor to be enrolled in clinical trials^198^. Compounds such as KU-55933 and KU-60019 have been shown to inhibit ATM *in vitro*, but their moderate potency, low water solubility, and low oral bioavailability have limited their further development^198^. AZD0156 was discovered after the chemical optimisation of a series of novel ATM inhibitors. Due to its high selectivity and excellent physicochemical properties, AZD0156 has become a good candidate for clinical drug development^198^. In preclinical trials, AZD0156 enhanced the usefulness of the PARP inhibitor olaparib in lung, gastric, and breast cancer cell lines^199^. In addition, AZD0156 increased radiation sensitivity in a mouse model of glioma^200^. Based on good preclinical anti-tumour activity, phase I evaluation of AZD0156 is currently underway, and further evaluation of its efficacy in combination with other drugs is planned.

#### AZD1390

AZD1390 is an ATM inhibitor with an oral activity that can penetrate the central nervous system. Compared to other PIKK family-related enzymes, AZD1390 has significant selectivity for ATM^202^. Prior to this, AZD0156 was clinically developed for the treatment of systemic tumours. However, as a matrix for human efflux transporters, AZD0156 is unlikely to significantly cross the BBB. AZD1390 was discovered during the optimal design of compounds using *in vitro* MDCK efflux transporter assays^201^. As a weak substrate of the transporter, it has significant penetration across the BBB, thus showing good therapeutic potential for central nervous system malignancies. AZD1390 enhances radiosensitization in multiple preclinical cancer cell types, making it a promising antitumor therapy. GBM is the most universal and potentially fatal type of brain tumour caused by malignant glial cells, and AZD1390 is presently in phase I clinical research phase of combined therapy with radiotherapy for GBM (NCT03423628). In addition, BRCA1/2 is a gene involved in the HR pathway. Research has shown that the combination of AZD1390 and EZH2 inhibitors has a synthetic lethal effect in the treatment of BRCA1-deficient breast tumors^202^. Thus, synergistic interactions can serve as an effective model for cancer treatment.

#### Peposertib/M3814

Although the older generation of DNA-PK inhibitors showed some efficacy, their selectivity was limited due to their structural similarity to PI3Ks. Peposertib is a new generation of oral and highly potent DNA-PK inhibitor^203^. In preclinical studies, peposertib effectively inhibited radiation-induced NHEJ repair when combined with radiotherapy, thereby enhancing the malignant cells’ reaction to radiation^204^. Furthermore, it has shown synergistic antitumor effects in NSCLC models when used in combination with the chemotherapeutic drugs paclitaxel and etoposide^205^. The good preclinical antitumor activity provides a theoretical basis for the follow-up clinical trials of peposertib. Peposertib was studied in a phase I study in combination with radiotherapy. There were 12 patients developing stable disease, and the RP2D was 400 mg twice daily (BID)^206^. At present, the combination of capecitabine and radiotherapy in the treatment of rectal cancer has not entered the Phase II trial, and the Phase Ib part has been completed (NCT03770689). Anyway, other studies combining M3814 with radiotherapy and chemotherapy are still ongoing.

#### CC-115

CC-115 is a dual inhibitor of DNA-PK and mTOR with IC_50s_ of 13 nM and 21 nM, respectively^207^. Initial clinical performance of CC-115 was evidenced in 8 patients with *ATM* deletion/mutation with relapsed/refractory CLL/SLL, and these patients were then enrolled in a larger phase I study^208^. In this broader Phase I study, the efficacy of CC-115 in patients with hematological malignancies or solid tumours was evaluated^209^. CC-115 displayed surprising efficacy in cervical squamous cell carcinoma, Ewing sarcoma, GBM, and castration-resistant prostate cancer, with stable disease rates of 53%, 22%, 21%, and 64%, respectively. Furthermore, a group of CLL/SLL patients treated with CC-115 showed promising clinical outcomes. 38% of patients with CLL had a partial response, and 25% of patients with SLL had stable disease. CC-115 is well tolerated and has similar toxicity to mTOR inhibitors. Therefore, CC-115 (10 mg BID orally) is an excellent regimen for anti-cancer therapy^209^. The II clinical trial of CC-115 combined with other drugs for GBM patients is currently underway (NCT02977780).

### mTOR inhibitors

**Figure 7.**
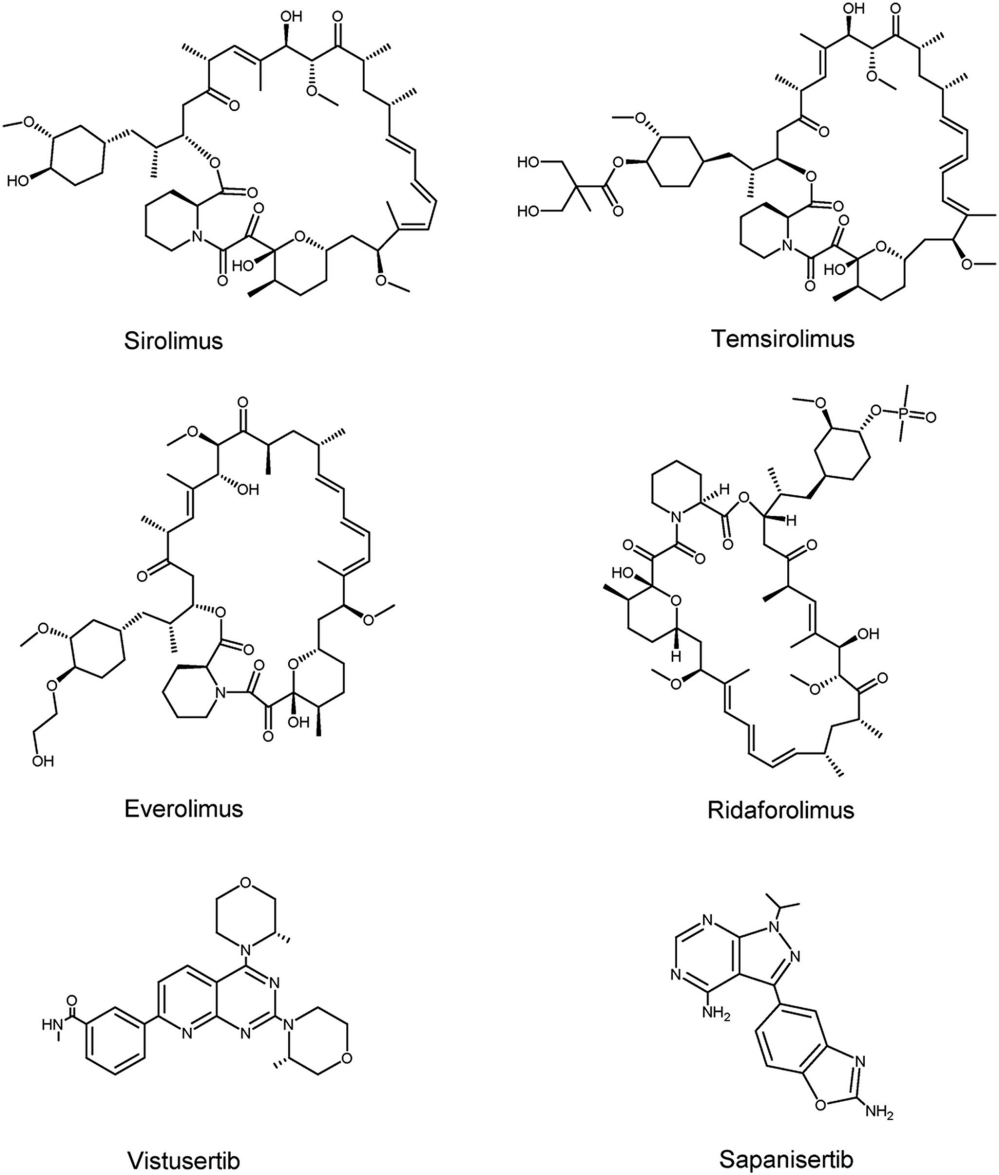
Structures of PIKK family members mTOR inhibitors.

Drug resistance in targeted mTOR therapy can emerge through a range of mechanisms, including inadequate mTORC1 function suppression, negative feedback loop inhibition, and compensatory pathway activation due to mTORC1 inhibition^210^. Rapamycin and its analogs are the first generation of mTOR inhibitors that selectively inhibit mTORC1 activity. However, rapamycin can inhibit the mTORC1 substrate S6K1, but cannot completely inhibit the phosphorylation of 4E-BP1, resulting in the inability to effectively block the inhibition of tumour growth^211^. Furthermore, S6K1 can phosphorylate insulin receptor substrate 1 (IRS-1), which is part of the negative feedback loop^179^. S6K1 also phosphorylates mSIN1, a component of mTORC2, at T86 and T398 and has a certain inhibitory effect on MTORC2-mediated AKT activation^212^. Since mTORC1 is the downstream target of several cancer-related pathways, inhibition of a single target can activate the compensation pathway, thereby decreasing the effectiveness of the cancer^210^. Based on this, developers have developed a series of mTORC1/mTORC2 inhibitors and PI3K/mTOR inhibitors. While these drugs are effective in overcoming drug resistance, they can be detrimental to normal cells and lead to severe toxicity, which may restrict their clinical use^213^. Due to the presence of multiple feedback loops and compensatory pathways, the effects of mTOR combined with other drugs are currently being explored. The structures of mTOR inhibitors are shown in Figure 7.

#### Rapamycin/sirolimus

Rapamycin, commonly known as sirolimus, has immunosuppressive and anti-proliferative effects, making it effective in the treatment of organ transplant rejection and cancer^214^. Rapamycin does not directly suppress mTOR function, instead, it binds to the intracellular FK506-binding protein 12 (FKBP12) to create the FKBP12-rapamycin complex, which subsequently binds to the FKBP12-rapamycin-binding (FRB) structure of the mTOR region^215^. Rapamycin insertion into the cavity produced by the FKBP12 and FRB domains changes the conformation of mTOR, limiting mTORC1 action allosterically^216^. Due to the insensitivity of mTORC2 to rapamycin, prolonged exposure to rapamycin can reduce the level of mTORC2. Therefore, it is believed that rapamycin inhibits the phosphorylation of mTOR substrates by binding to mTORC1 rather than mTORC2^217^. Rapamycin is limited in clinical practice due to its unfavourable physicochemical properties and pharmacokinetic characteristics. Hence, many structural changes and transformations of sirolimus have been carried out with the aim of finding more efficient rapamycin analogs. Furthermore, nab-sirolimus (ABI-009) is an innovative mTOR inhibitor, a suspension of sirolimus albumin-bound nanoparticles for injection. Due to its unique dosage form, nab-sirolimus has a superior pharmacokinetics profile, higher tumour tissue drug exposure, stronger target cell inhibition, and better safety profile than other marketed mTOR inhibitors^218^. In a phase II trial of advanced malignant PEComa, nab-sirolimus monotherapy resulted in an ORR of 39%, a median PFS of 10 months, and a median overall survival of 48 months^219^. Based on the drug’s excellent efficacy in this trial, the FDA has approved it for intravenous injection in adult patients with malignant PEComa^220^.

#### Temsirolimus/CCI-779

Temisirolimus, an ester analogue of rapamycin, was approved by the FDA in 2007 for the treatment of advanced renal cell carcinoma (RCC)^221^. Temisirolimus’ efficacy in the treatment of various malignant cancers in combination with other chemotherapy medicines is still being evaluated. In patients with first-relapse rhabdomyosarcoma (RMS), a phase II trial was scheduled to estimate the feasibility of bevacizumab or temsirolimus in conjunction with chemotherapy. The ORR of the bevacizumab and temsirolimus groups was 28% and 47% respectively, and the 6-month event-free survival was 54.6% and 69.1%. Due to the superior efficacy of the temsirolimus group, temsirolimus will be recommended as the bioactive agent for RMS^222^. Tesirolimus plus erlotinib was well tolerated in patients with solid tumours, of whom nine patients (82%) had stable disease, establishing the recommended RP2D^223^. In one trial, temsirolimus was coupled with the AKT inhibitor perifosine to treat relapsed GBM. There have been no previous studies that combined these two kinds of medicines with the goal of efficiently inhibiting the PI3K/AKT signalling cascade. The MTD of the combination was established by the trial to be 115 mg of temsirolimus weekly and 100 mg of perifosine daily. Notably, the temsirolimus dose was more than four times higher than the FDA-approved amount for RCC (25 mg per week). The reasons why large dosages of temsirolimus were tolerated in this trial are unknown; nonetheless, additional pilot studies are planned^224^.

#### Everolimus/RAD-001

Everolimus, a 40-*O*-(2-hydroxyethyl) derivative of rapamycin, was granted FDA approval in 2019 for the treatment of advanced postmenopausal HR^+^/HER2^−^ breast cancer in women, pancreatic-derived progressive neuroendocrine tumours, renal vascular smooth muscle lipoma, RCC, and other indications^225^. In phase II randomised trial, everolimus plus letrozole was used to treat women with advanced premenopausal breast cancer who were HR^+^/HER2^−^. Patients in the everolimus plus letrozole group received longer median PFS than those in the letrozole alone group (19.4 months vs. 12.9 months), indicating that the combination was beneficial^226^. Similarly, everolimus combined with an aromatase inhibitor (AI) has shown promising therapeutic activity in patients suffering from breast cancer. The median PFS in the everolimus plus AI group and the AI monotherapy group was 11.0 months and 7.2 months respectively, with ORRs of 22.4% and 19.2% respectively^227^. When everolimus was combined with bevacizumab to treat patients with the RCC form of metastatic cancer, the median PFS was 13.7 months with an ORR of 35%. The combination’s robust activity supports this regimen as the standard choice for patients with RCC^228^. Overall, everolimus has displayed considerable potential in various cancer treatments, and the efficacy of different combination regimens of everolimus is currently being further explored.

#### Ridaforolimus/deforolimus/MK-8669

Ridaforolimus is a sirolimus analog that was initially used to treat soft tissue and osteosarcoma before being extended to treat a wide range of solid and hematological malignancies^229^. A phase III trial evaluated the maintenance of disease control in advanced sarcoma with ridaforolimus. The median PFS was 17.7 weeks for ridaforolimus and 14.6 weeks for placebo. However, serious adverse effects (grade > 3) were more common with ridaforolimus (64.1% vs. 25.6%)^230^. Due to its poor risk-benefit profile, the US FDA has not approved ridaforolimus for the treatment of sarcoma. Ridaforolimus underwent subsequent clinical trials for the treatment of various malignancies, but its safety still restricts its clinical application. In patients with advanced endometrial cancer, single-agent ridaforolimus displayed anticancer activity and tolerable tolerability, with 13 patients (29%) attaining clinical benefit and a 6-month PFS rate of 18%^231^. Based on the positive clinical outcomes of this trial, a follow-up phase II trial was conducted in patients with endometrial cancer^232^. The median PFS for the ridaforolimus group was 3.6 months and 1.9 months for the control group (progestin or chemotherapy). However, the proportion of patients in the ridaforolimus group who discontinued treatment due to AE was 33%, compared to 6% in the control group, with typical grade 3 toxicities being hyperglycaemia, anaemia, and diarrhoea^232^. Thus, oral ridaforolimus has strong toxicity in advanced endometrial cancer. Currently, ridaforolimus has stopped development.

#### Vistusertib/AZD2014

Vistusertib is an ATP-competitive mTOR inhibitor that inhibits the mTORC1 and mTORC2 complexes^233^. Intermittent dosing regimens induce tumour regression when vistusertib is combined with fulvestrant, making the compound an ideal candidate for clinical combination with endocrine therapy^233^. According to one study, when compared to single-agent anastrozole, combining vistusertib with anastrozole significantly improved clinical benefit in patients with endometrial cancer. The ORRs were 17.4% and 24.5% respectively, and the PFS were 1.9 and 5.2 months respectively, with manageable toxicity^234^. A phase II randomised study compared vistusertib with everolimus in patients with mRCC. The PFS for AZD2014 and everolimus was 1.8 months and 4.6 months, respectively, with 35% and 48% of patients experiencing grade 3–4 adverse effects. As a result, while vistusertib is less hazardous, its anticancer activity is inferior to that of everolimus^235^. When treating patients with polymorphic GBM, vistusertib plus temozolomide resulted in a 6-month PFS rate of 26.6%. The combination test demonstrates tentative efficacy and is relatively safe^236^. The median PFS for both types of patients was 5.8 months when vistusertib plus paclitaxel was used in patients with ovarian cancer and NSCLC. Due to tolerability concerns, patients with NSCLC must adjust their dose accordingly, and the toxicity is manageable^237^. A recent phase I/II study found that vistusertib was well tolerated in paediatric patients, indicating that it should be investigated in the appropriate paediatric therapy route^238^.

#### Sapanisertib/INK128/MLN0128/TAK-228

Sapanisertib is an oral, potent ATP-competitive mTOR inhibitor^239^. In a phase I study of East Asian patients with advanced nonhematologic malignancies, sapanisertib revealed good tolerability and limited anticancer efficacy. Clinical benefit rates with once-daily (QD) and once-weekly doses were 45% and 67% respectively, with RP2D determined to be 3 mg QD^240^. Subsequently, clinical research was conducted on the combination of sapanisertib and other anticancer therapies. Both combinations of sapanisertib with alisertib or paclitaxel were well tolerated in patients with solid tumours, implying that further research into the combination’s efficacy is required^240^^,^^241^. A phase II trial looked into the effectiveness of sapanisertib plus fulvestrant in breast cancer patients. When compared to single-agent fulvestrant, sapanisertib plus fulvestrant significantly increased PFS by 3.5 months and 7.2 months, respectively. However, due to the increased toxicity produced by combination therapy, future research has been limited^242^. Moreover, research has indicated that combining tamoxifen and sapanisertib is a promising neoadjuvant modality for individuals with ER^+^ breast cancer^243^. Currently, CB-839 HCl combined with sapanisertib treatment for advanced NSCLC in phase I/Ib trials is still recruiting patients(NSCLC).

## AKT

AKT is a class of serine/threonine kinase that belongs to the AGC kinase family, also widely recognised as PKB^244^. The Akt family consists of three members: Akt1 (PKB α), Akt2 (PKB β), and Akt3 (PKB γ). The three AKT isotypes have similar structures, including the PH domain, the central kinase domain, and the carboxyl-terminal regulatory domain containing hydrophobic motifs^245^. Although the three subtypes have similar structures, they can perform different functions in cancer and physiology. AKT1, as a growth inducer, promotes tumour cell growth but damages metastasis, while AKT2 can increase tumour cell invasion and metastasis^246^. Thus, AKT1 is hypothesised to act as an invasion suppressor in the early stages of the disease, while AKT2 promotes invasion in the advanced stages of the disease^247^. AKT3 is the least studied and has been proven to be closely linked to the progression of triple-negative breast cancer (TNBC)^248^. The subtype-specific function of AKT remains controversial, and the mechanisms by which it impacts cancer progression require further investigation. Nonetheless, AKT remains a key target for the treatment of cancer.

AKT is an indispensable part of PI3K-mediated signalling and its activation can phosphorylate a variety of kinases and transcription factors, thereby affecting cellular functions. For example, the phosphorylation of TSC2 by AKT can inhibit the GTPase activity of the TSC1/TSC2 complex, leading to the accumulation of Rheb-GTP and subsequent mTORC1 activation^179^. AKT phosphorylates Glycogen synthase kinase-3β to facilitate glucose metabolism^249^. AKT is able to activate the inhibitor of kappa B kinase (IKK), which is involved in the modulation of IκBα. Phosphorylated IκBα is rapidly degraded to release NF-κB, and the released NF-kB translocates to the nucleus and induces the expression of target genes, thus playing an important role in promoting tumour survival^3^. AKT phosphorylates forkhead box protein O1, inhibiting its nuclear translocation and preventing its transcriptional activation^250^. AKT phosphorylates the ubiquitin ligase MDM2 (mouse double microgene 2) and translocates it to the nucleus to bind to p53, thereby affecting cell survival by increasing the degradation of the p53 protein^251^. AKT activation is closely related to the development and progression of various tumours, so suppressing AKT may be an excellent therapeutic approach.

## AKT inhibitors

Overactivation of the PI3K/AKT pathway is common in many different types of cancer, and AKT is a critical hub of signalling pathways in cancer, making targeting AKT an appealing anticancer tactic^41^. It can be challenging to develop specific inhibitors because the sequence identity of the kinase domains of the three isoforms is more than 80%, varying only at the PH structural domain and kinase structural domain junctions^252^. Drugs are classified as allosteric inhibitors or ATP-competitive inhibitors based on the structure of AKT and the difference in binding domain with the inhibitor^253^ (Table 3). There are few reports of ATP-competitive inhibitors in early development, because the structural similarity of the ATP-binding site allows them to both target AKT and inhibit other kinases of the AGC kinase family with high AKT homology. Thus, lack of kinase selectivity makes them prone to off-target toxicity^254^. Many AKT inhibitors exhibit limited anticancer activity as clinically indicated monotherapies; therefore, ongoing clinical trials of AKT inhibitors are focused on exploring their potential to improve standard cancer therapy. In addition, some AKT inhibitors such as miransertib have shown excellent efficacy in the treatment of cutaneous leishmaniasis or Proteus syndrome^255^.

**Table 3.** A summary of major AKT inhibitors.

Inhibitor	Compound	Inventor	IC_50_ (nmol/L)	Phase	Clinical
Allosteric inhibitor	MK-2206	Merck	Akt1/2/3: 8/12/65	II	NCT01277757
	BAY 1125976	Bayer	Akt1/Akt2: 5.2/18	Terminated	
	TAS-117	Takeda	Akt1/Akt2/Akt3: 4.8/1.6/44	II	NCT03017521
	Miransertib(ARQ-092)	ArQule	Akt1/Akt2/Akt3: 2.7/14/8.1	II	NCT03094832
	ALM301	Almac Discovery Ltd	Akt1/2: 125/95	Preclinical	
Competitive inhibitor	Capivasertib(AZD5363)	AstraZeneca	Akt1/Akt2/3: 3/8/8	III-Ongoing	NCT03997123
	Afuresertib(GSK2110183)	GlaxoSmithKlie	Akt1/Akt2/Akt3: 0.08/ 2/ 2.6 (Ki)	II	NCT01532700
	Uprosertib(GSK2141795)	GlaxoSmithKlie	Akt 1/2/3: 180/328/38	II	NCT01907815
	Ipatasertib(GDC0068)	Roche	Akt1/2/3: 5/18/8	III-Ongoing	NCT03072238
	NTQ1062	Zhengda Tianqing	Akt1/Akt2/Akt3: 0.4 /6.3/0.1	I	CTR20211999

### Allosteric inhibitors

**Figure 8.**
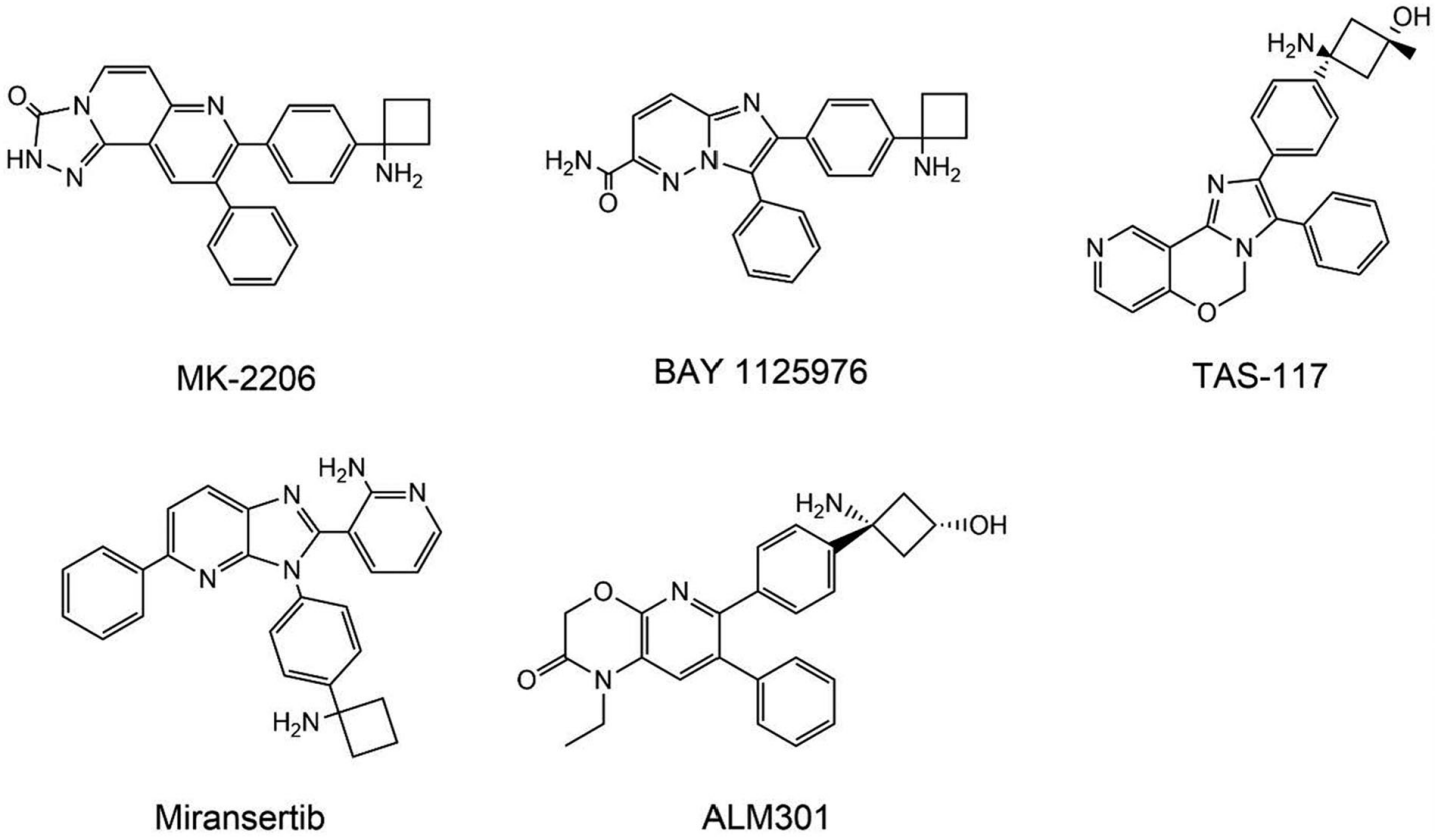
Structures of AKT allosteric Inhibitors.

Allosteric inhibitors bind to a specific hydrophobic area at the intersection of the PH domain and the kinase domain, rather than the ATP binding site or the PH domain. As a result, a stable AKT "PH-in" conformation is formed, the phospholipid binding site is blocked, AKT activation is obstructed, and the selectivity of the three AKT isoforms is achieved by utilising the diversity and allosteric sites of the amino acid sequence of the AKT hinge region^256^. Allosteric modulators have several advantages, including greater specificity, fewer adverse effects, and lower toxicity. The structures of AKT allosteric inhibitors are shown in Figure 8.

#### MK-2206

MK-2206 is the first small molecule allosteric AKT inhibitor to enter clinical trials. MK-2206 inhibits AKT autophosphorylation at Thr 308 and Ser 473, which results in anticancer action^257^. In the phase II study, MK-2206 had little efficacy in advanced breast cancer patients with genetic alterations or deletions^258^. The ORR and 6-month PFS were 5.6% for the *PIK3CA*/*AKT1* mutation group, while the ORR was 0% and the 6-month PFS was 11% for the *PTEN* deletion/mutation group. Toxicity and tumour-related features reduced dose delivery, leading to insufficient tumour target inhibition and ultimately limited anticancer efficacy^258^. Likewise, MK-2206 monotherapy showed modest activity in renal cell carcinoma and in lymphoma^259^^,^^260^. When MK-2206 was combined with bendamustine and rituximab to treat patients with CLL, the ORR was 92% and the median PFS was 16 months, making it a promising novel therapeutic combination for CLL^261^. In patients with *HER2* amplified solid tumours, MK-2206 was coupled with paclitaxel and trastuzumab, and 10 patients (63%) responded with a median response duration of 6 months^262^. Although some activity signals were observed in the combination therapy of MK-2206, no new research has been conducted on MK-2206 due to efficacy and tolerance issues.

#### BAY 1125976

BAY 1125976 is a novel, orally active, and allosteric AKT1/2 inhibitor. BAY 1125976 inhibits the expansion of multiple human cancer cells, with striking activity in breast and prostate cancer cell lines, paving the way for clinical development^263^. In the phase I study, 30 of the 78 patients enrolled were stable. In 43 patients treated with RP2D, the clinical benefit rate was 27.9%^264^. BAY 1125976 was well tolerated; however, clinical tumour responses were limited. At present, the development of BAY 1125976 is still in phase I testing.

#### TAS-117

TAS-117 is an oral allosteric AKT inhibitor. Preclinical evaluations show that TAS-117 can induce autophagy and apoptosis, and can significantly inhibit tumour growth in multiple myeloma cells^265^. AS-117 combined with the dual EZH2/1 inhibitor UNC1999 and the FGFR1-4 inhibitor futibatinib respectively has synergistic anti-tumour effects in cancer models^266^^,^^267^. At present, TAS-117 is in human clinical research. In a phase II trial in patients with advanced solid malignancies with PI3K/AKT pathway abnormalities, TAS-117 showed anti-tumour activity in patients with *PIK3CA* H1047R and *AKT1E17K*-mutated breast cancer and *PIK3CA* E545K-mutated ovarian cancer. The ORR was 8%, and the median PFS was 1.4 months^268^. TAS-117 is being studied in a phase II trial in patients with advanced solid tumours possessing germline *PTEN*-inactivating mutations (NCT04770246).

#### Miransertib/ARQ-092

Miransertib is a novel oral AKT inhibitor that blocks membrane translocation of inactive AKT and dephosphorylates active AKT, thereby disrupting AKT activity^269^. Miransertib was initially used to treat human malignant tumours, but in recent years it has primarily been used to treat a variety of rare disorders, including PIK3CA-related overgrowth spectrum caused by somatic activating mutations in PIK3CA and Proteus syndrome caused by somatic activation in AKT1^255^,^269^. As a result, targeting the PI3K pathway offers promise for treating such diseases, and several clinical studies have been performed with miransertib to treat related diseases. Furthermore, miransertib has a curative impact on visceral and cutaneous leishmaniasis^270^.

#### ALM301

Based on the Bicyclic pyridine and oxazidone core, which has demonstrated remarkable biochemical efficacy in drug development, a novel variant of the AKT inhibitor ALM301 has been developed in recent years^271^. ALM301 effectively and selectively inhibits AKT1 and AKT2, with IC_50_ values of 125 nM and 95 nM, respectively^271^. ALM301 has significant antiproliferative effects on sensitive cell lines and exhibits excellent efficacy in both single and combination therapy in rats, providing important clues for us to further explore the potential of ALM301 in clinical treatment.

### Competitive inhibitors

**Figure 9.**
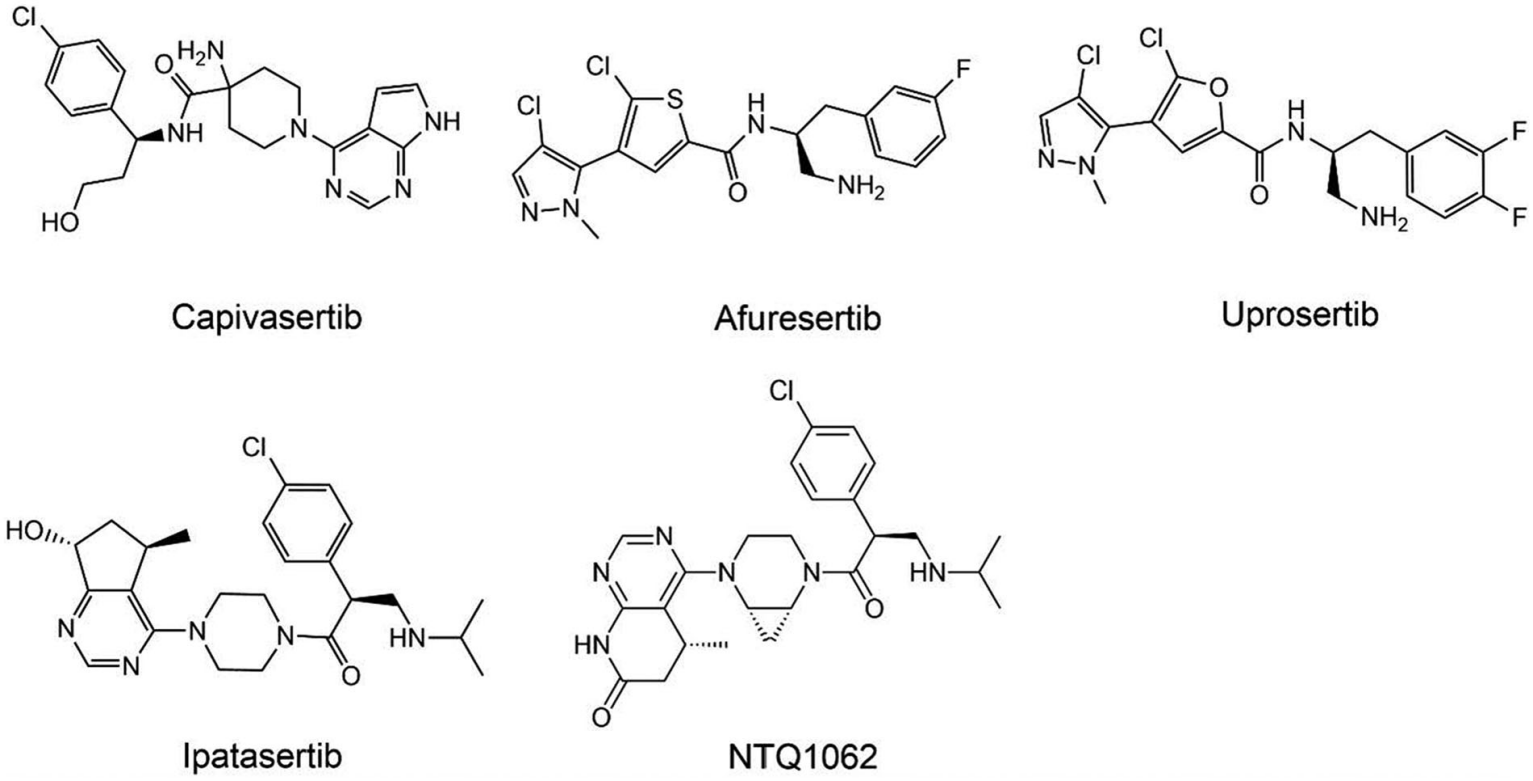
Structures of AKT competitive inhibitors.

ATP-competitive inhibitors compete with ATP for the AKT kinase domain active sites, preventing activated AKT from phosphorylating its downstream target proteins^256^. Since the catalytic domains are so similar, ATP-competitive inhibitors are ineffective against AKT isozymes and very marginally selective for closely related kinases^254^. A future goal is to create ATP-competitive inhibitors that specifically inhibit specific subtypes of AKT. The following is a list of some of the ATP-competitive inhibitors that have reached the clinical stage. The structures of AKT competitive inhibitors are shown in Figure 9.

#### Capivasertib/AZD5363

Capivasertib is a potent oral ATP-competitive inhibitor developed by AstraZeneca that can effectively inhibit all isoforms of AKT. Capivasertib has a limited single-agent effect, but it is more effective when combined with traditional chemotherapy, and can be used as a sensitiser for tumour chemotherapy^272^. Several trials have confirmed the effectiveness of combination drugs. Capivasertib has shown promising efficacy in breast cancer and has significant activity against AKT1-E17K-mutated cancers. The median PFS of TNBC patients receiving treatment with capivasertib and paclitaxel was 5.9 months. In *PIK3CA*/*AKT1*/*PTEN*-altered tumours, this regimen was significantly beneficial, with a median PFS of 9.3 months^273^. When capivasertib was coupled with fulvestrant in patients with resistant breast cancer, the capivasertib group considerably surpassed the placebo group in terms of median PFS (10.3 months and 4.8 months, respectively). As a result, a relevant phase III trial is planned^274^. Furthermore, AKT signalling overexpression causes radioresistance in oral squamous cell carcinoma (OSCC) cells, and capivasertib encapsulated in new nanoparticles can sensitise radioresistant OSCC cells to IR^275^. Therefore, combining Capivasertib with nanoparticles is an effective strategy for treating OSCC patients. Currently, research on the combination therapy of Capivasertib is ongoing.

#### Afuresertib/GSK2110183

Afuresertib is an oral ATP-competitive inhibitor that inhibits all isoforms of AKT. Afuresertib showed single-agent activity against hematological malignancies, particularly Multiple myeloma (MM), with 3 of 34 MM patients achieving PR (9%), and the others maintaining long-term disease stability^276^. Afuresertib plus carboplatin and paclitaxel is an attractive tactic for treating ovarian cancer. The ORR and median PFS were 32% and 7.1 months, respectively^277^. In patients with solid tumours, continuous daily dosing of afuresertib plus trametinib was poorly tolerated. Therefore, further study of intermittent dosing regimens is required^278^. In the phase II study in patients with CLL, the ORR of afuresertib in combination with ofatumumab was 50%, with a median PFS of 8.5 months^279^. Although combination therapy has certain activities and good tolerability, it cannot demonstrate greater benefits than single-drug treatment with afuresertib. At present, new drug combinations related to afuresertib are under intensive research.

#### Uprosertib/GSK2141795/GSK795

Uprosertib is an orally active N-alkylpyrazole AKT inhibitor that induces tumour cell growth arrest *in vitro*, enhances cisplatin-induced apoptosis, and reduces tumour volume *in vivo* when combined with platinum-based cytostatics^280^. Therefore, AKT inhibition has clinical therapeutic potential for platinum-resistant cancers. Clinical trials have used dual suppression of PI3K and RAS signalling. In patients with leukaemia, endometrial cancer, and melanoma, uprosertib in combination with trametinib displayed modest clinical activity, indicating the need for more effective therapeutic options^281–283^. When trametinib was combined with uprosertib in patients with recurrent cervical cancer, 8 of 14 patients had stable disease, and 3 progressed to the best response. This combination is feasible in cervical cancer patients, but dose reductions are still required to reduce adverse events^284^. The investigators will further explore more effective combination therapy strategies related to uprosertib.

#### Ipatasertib/GDC0068/RG7440

Ipatasertib is a highly selective oral ATP-competitive inhibitor that targets all subtypes of AKT^285^. At present, ipatasertib is mainly developed for the treatment of solid tumours, and a number of clinical trials have been conducted to evaluate its efficacy. The combination of ipatasertib and abiraterone has entered a phase III clinical study in patients with *PTEN*-deficient mCRPC^287^. Ipatasertib inhibits PI3K-AKT-mTOR signalling, whereas abiraterone suppresses androgen signalling. The combination therapy increased radiographic PFS by up to 18.5 months^286^. Patients with TNBC were enrolled in the phase II trial of ipatasertib plus paclitaxel^286^. When contrasted to the placebo group, the ipatasertib group had a slightly longer median PFS (6.2 months vs. 4.9 months). This was also true in 48 patients with *PTEN*-low tumours (6.2 months vs. 3.7 months)^287^. In a nutshell, ipatasertib has surprising potential in the treatment of breast and prostate cancers.

#### NTQ1062

NTQ1062 was discovered based on the ipatasertib structural skeleton by optimising hinge adhesive regions and connections^288^. NTQ1062 showed good potency and pharmacokinetic properties with IC_50_ values of 0.4, 6.3, and 0.1 nM for Akt1/Akt2/Akt3, respectively^288^. Given that NTQ1062 has demonstrated promising anticancer efficacy *in vitro* and *in vivo*, phase I clinical studies of NTQ1062 are currently underway (CTR20211999).

## Conclusions and future outlooks

In the past few years, PI3K and AKT have become the research objects of targeted cancer therapy due to their crucial roles in the PI3K/AKT/mTOR signalling cascade. At present, a variety of inhibitors have entered clinical research, and they have shown good prospects in clinical trials. Among them, five drugs targeting PI3K have been approved by the FDA for marketing. PIKK and PI3K are two related kinase families with identical origins and very similar kinase structural domains but distinct characteristics. Extensive research on these kinases has demonstrated that inhibiting PIKK family kinases is useful in overcoming resistance to cancer therapy, and so the design and implementation of PIKK inhibitors could be a novel technique for effective cancer and other illness treatment. LY294002, the first-generation PI3K inhibitor, may efficiently inhibit PI3K, mTOR, DNA-PK, and ATM. Wortmannin can inhibit all four isoforms of PI3K, as well as mTOR and DNA-PK. Due to their poor physicochemical properties or adverse effects, both inhibitors have been excluded from further biological evaluation and clinical trials. They are, however, useful chemical tools for the development of other PI3K and PIKK inhibitors. In recent years, as the understanding of PIKK family kinases has deepened, inhibitors related to DNA damage response and mTOR inhibitors have started to appear prevalently in the public spotlight. A broad number of ATM, ATR, DNA-PK, and mTOR inhibitors have entered clinical trials, but only mTOR has an authorised medication on the market.

At present, safety and resistance concerns are still key obstacles to the development of this category of medications, making it difficult to make a splash in the oncology drug market. Cancers can rapidly adapt to a single chemotherapeutic drug due to the reactivation of blocked pathways or the activation of parallel signalling pathways caused by the initiation of compensatory mechanisms. Combining additional medications is thus regarded as a very interesting research avenue. These medications are typically administered in two ways: orally and intravenously. To improve anti-cancer efficacy, the most appropriate drug delivery method must be selected based on the pharmacokinetic characteristics of different drugs. Furthermore, nanodrug delivery systems (NDDS) can help overcome delivery barriers, reduce chemotherapy side effects, and achieve precision treatment, making NDDS an effective treatment strategy when combined with chemotherapy drugs. In addition, compared to continuous administration, intermittent administration of PI3K-related inhibitors is not only well tolerated but also more effective as an anticancer therapy compared to continuous dosing. Tumour invasion and progression are of considerable complexity. In recent years, multi-targeted therapy has been considered an attractive means of treating cancer to circumvent drug resistance and improve the safety of treatment. For example, PI3K/mTOR, PI3K/HDAC, PI3K/AKT/mTOR, and PDGFR/PI3K/EGFR/VEGFR2 multi-target inhibitors were developed with the hope of effectively improving anti-tumour activity. As approval of anticancer drugs becomes more difficult, developers are forced to reconsider safety issues, thus facilitating the re-optimisation of inhibitors. The safety and target selectivity of drugs can be enhanced through the development of combination therapies, optimisation of drug delivery routes and regimens, and the design of drug structures with the aim of improving the safety and resistance of drugs.
